# On an enhancement of RNA probing data using information theory

**DOI:** 10.1186/s13015-020-00176-z

**Published:** 2020-08-07

**Authors:** Thomas J. X. Li, Christian M. Reidys

**Affiliations:** 1 Biocomplexity Institute & Initiative, University of Virginia, 995 Research Park Blvd, Charlottesville, VA 22911 USA; 2 Department of Mathematics, University of Virginia, 141 Cabell Dr, Charlottesville, VA USA

**Keywords:** RNA structure, Chemical probing, Rényi-Ulam game, Information theory, Primary 92E10, Secondary 94A15, 92B05

## Abstract

Identifying the secondary structure of an RNA is crucial for understanding its diverse regulatory functions. This paper focuses on how to enhance target identification in a Boltzmann ensemble of structures via chemical probing data. We employ an information-theoretic approach to solve the problem, via considering a variant of the Rényi-Ulam game. Our framework is centered around the ensemble tree, a hierarchical bi-partition of the input ensemble, that is constructed by recursively querying about whether or not a base pair of maximum information entropy is contained in the target. These queries are answered via relating local with global probing data, employing the modularity in RNA secondary structures. We present that leaves of the tree are comprised of sub-samples exhibiting a distinguished structure with high probability. In particular, for a Boltzmann ensemble incorporating probing data, which is well established in the literature, the probability of our framework correctly identifying the target in the leaf is greater than $90\%$.

## Background

Computational methods for RNA secondary structure prediction have played an important role in unveiling the various regulatory functions of RNA. In the past four decades, these approaches have evolved from predicting a single minimum free energy (MFE) structure [1, 2] to Boltzmann sampling an ensemble of possible structures [3, 4]. Despite its success in a wide range of small RNAs, these thermodynamics-based predictions are by no means perfect.

In parallel, experiments by means of chemical and enzymatic probing have become a frequently used technology to elucidate RNA structure [5–7]. The basic idea of these probing methods is to use chemical probes that react differently with paired or unpaired nucleotides. The binding sites can later be detected by biochemical techniques, such as selective $2'$-hydroxyl acylation with primer extension (SHAPE) [7, 8], which yield reactivities at nucleotide resolution. To some extent, these reactivities provide information concerning single-stranded or double-stranded RNA regions. However, the reactivity does not unambiguously determine a specific position to be unpaired or paired [9]. While high SHAPE reactivity matches well with unpaired nucleotides, medium reactivity could correspond either to paired or unpaired nucleotides depending on various factors, such as the RNA structure itself or the experimental conditions. Recent advances focus on the development of thermodynamics-based computational tools that incorporate such experimental data as soft constraints to handle the ambiguity [7, 10, 11].

While the use of probing data has significantly improved the prediction accuracy of in silico structure prediction for several classes of RNAs [12], these methods have not solved the folding problem for large RNA systems, such as long non-coding RNAs (lncRNAs, typically 200–20k bases). The reason is that the footprinting data is *one-dimensional*, i.e. it does not identify base pairing partners of a given nucleotide. In particular, probing data alone cannot distinguish short-range and long-range base pairings. For long RNAs, the existence of the latter, however, has been shown experimentally [13] as well as theoretically [14, 15]. Thus, even combined with experimental data, there are still numerous RNA folds consistent with the probing data.

Based on chemical probing, Sanbonmatsu et al. [16] developed a fragmentation method for determining the secondary structure of lncRNAs in the wet lab. Their approach applies chemical probing of the entire RNA, followed by parallel probing of certain overlapping fragments. Regions of each fragment exhibiting similar probing profiles are folded independently, and combined in order to obtain the entire structure. Although the method has been successfully applied to identify the structures of several lncRNAs [16, 17], their choice of fragments is empirical, which hinders its application to longer RNA sequences [17].

In the following, as in [16], we shall stipulate $(*)$: in all probing experiments there exists a unique distinguished structure, the *target*. We furthermore assume that the collection of all possible structures is in thermodynamic equilibrium, i.e. a Boltzmann ensemble, and the target is contained in the ensemble. Hence, the problem of structure prediction gives rise to the following challenge:

1 $$\begin{aligned} {\textit{How to enhance target identification in a Boltzmann ensemble of structures?}} \end{aligned}$$

**Fig. 1:**
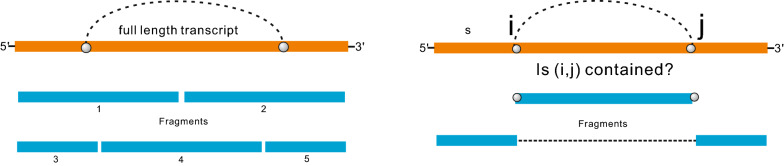
The fragmentation of Sanbonmatsu et al. [16] (LHS) and our approach (RHS). Their approach almost certainly “breaks” long-range base pairs (dotted arcs), while ours allows bases from two non-contiguous fragments to pair

In relation to [16], our approach to Problem 1 can be understood as well as outlined as follows: Sanbonmatsu employs *in parallel* localization of the chemical probing experiment via fragmentation. The latter are somewhat ad hoc and almost certainly “break” any long-range base pairs, see Fig. 1.

The novelty of this paper lies in a different, sequential fragmentation process, assuming $(*)$. Our input consists of the probing data of the entire sequence, giving rise to an augmented Boltzmann ensemble containing by construction the target (which of course is not known). Instead of a parallel fragmentation into subsequences, we “localize” differently, namely we successively ask whether a specific base pair is contained in the target or not. The particular base pair is identified using information theoretic properties of the Boltzmann ensemble. While we do not know the target explicitly, we can decide, with high accuracy, if it contains a particular base pair. Specifically, we cut the subsequence covered by the base pair, and glue the remainder at the cut-points. On the resulted two subsequences, our approach requires probing data to be generated from probing experiments. We then compare the additional probing data with the initial probing profile of the entire sequence. At a fundamental level, our fragmentation is different from Sanbonmatsu’s approach [16], in that we allow bases from two non-contiguous fragments to pair, see Fig. 1. As a consequence, our method is well suited to deal with the long-range base pairings, these being a prominent feature of RNA secondary structures [13, 14].

**Fig. 2:**
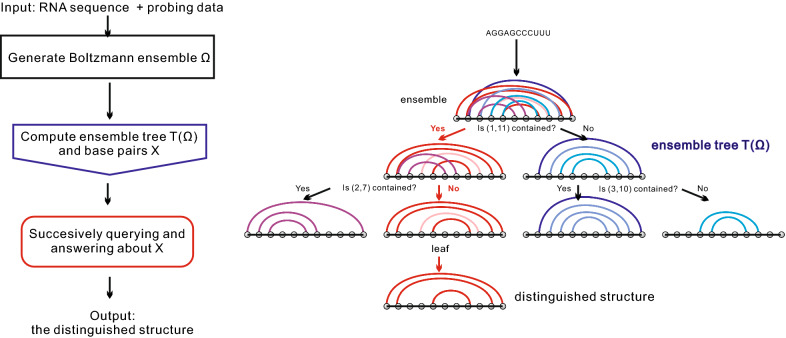
The workflow diagram (LHS) and schematic example (RHS) of our approach. LHS: we compute the ensemble tree, i.e., successively split the Boltzmann ensemble of structures into smaller sub-samples, by querying certain base pairs is contained in the target or not. RHS: the answers to the base-pair questions allow us identify a path (red) in the ensemble tree from the root to the leaf, which contains a distinguished structure

The answer to each question produces a split of the ensemble into two sub-samples, and we arrive at smaller sub-samples via successively querying and answering. We then establish that, after a few iterations, we arrive at a sample that contains a distinguished structure that, with high probability, coincides with the target. We illustrate the overall strategy in Fig. 2.

We formalize the above sequential process, by considering a variant of the Rényi-Ulam game, in which a player tries to identify an unknown object via asking yes–no questions [18, 19]. Our framework is centered around the *ensemble tree*, a hierarchical bi-partition of the input ensemble, whose leaves are comprised of sub-samples exhibiting a distinguished structure with high probability. Specifically, the ensemble tree is constructed by recursively querying about whether or not a base pair of maximum information entropy is contained in the target. We prove that the query of maximum entropy base pair splits the ensemble into two even parts and in addition provides maximum reduction in the entropy of the ensemble. These questions can be answered in the affirmative because of assumption $(*)$. They are answered via relating additional “local” probing data with the initial one, employing the modularity in RNA secondary structures. By this means, we identify the correct path in the ensemble tree from the root to the leaf.

The key result of this paper is that the probability of the ensemble tree correctly identifying the target in the leaf is greater than $90\%$, for the Boltzmann ensembles from random sequences of length 300, in “Target identification” section. To demonstrate the result, we firstly utilize a *q*-Boltzmann sampler with signature distance filtration, which is well suited for Boltzmann ensembles subjected to the probing data constraint [7, 11], see “The Boltzmann ensemble” section. Secondly, we consider the error rates arisen from answering the queries via probing data. We show that these error rates can be significantly reduced via repeated queries in “Target identification” section. Thirdly, in “Entropy” section, we prove that the leaf with low information entropy contains a distinguished structure. We present that, once in the correct leaf, the probability the distinguished structure being identical to the target is almost always correct. Fourthly, in “Robustness” section, we analyze the robustness of our approach. We demonstrate that the ensemble tree localizing the target with high fidelity is robust, across Boltzmann samples of different sizes and nucleotide compositions.

We would point out that the *q*-Boltzmann sampler is only required to benchmark our approach on random sequences, due to the absence of probing data. In application scenarios where chemical probing data is provided, our approach utilizes Boltzmann ensembles with soft constraints [7].

As proof of concept, we apply our approach to natural RNAs with SHAPE probing data and compute the distinguished structure from the ensemble tree to predict the accepted secondary structure, i.e., the target. We show in “Performance comparison” section that our approach improves the average prediction accuracy by $5\%$, compared with [8].

To summarize, the key points of our approach are:

1. our method is based on a Boltzmann sample and derives a sub-sample that contains the target with high probability,
2. the derivation is facilitated by means of the ensemble tree, and the identification of the correct path from root to leaf, is obtained by a variant of the Rényi-Ulam game,
3. the answers to the respective queries are inferred from chemical probing, by relating additional probing data to the initial data using modularity.

This paper is organized as follows: in “Methods” section, we introduce the main elements of our framework: the Rényi-Ulam game, the Boltzmann ensemble, base-pair queries and the ensemble tree. In “Path identification” section, we demonstrate how to integrate additional probing data with the initial ones allowing to answer the queries, thereby identifying the correct path. In “Results” section, we analyze the ensemble tree and present that our approach identifies the target reliably and efficiently. Finally, we discuss and integrate our results in “Discussion” and Conclusion” sections.

## Methods

### The Rényi-Ulam game

We now approach Problem 1 via the Rényi-Ulam game, a two-person game, played by a questioner (Q) and an oracle, (O). Initially O thinks of an integer, *Z*, between one and one million and Q’s objective is to identify *Z*, asking yes-no questions. O is allowed to lie at a rate specific to yes and no, respectively.

The Rényi-Ulam game has been extensively studied since the early works by Rényi and Ulam [18, 19], and has various applications such as adaptive error-correcting codes in the context of noisy communication [20, 21]. Depending on the respective application scenario, numerous variants of the Rényi-Ulam game have been considered, specifying the format of admissible queries or the way O lies [22, 23].

In what follows, we shall play the following version of the game: O holds a set of *bit strings*$y_1 y_2 \ldots y_l$ of finite length *l*, not every bit string being equally likely selected and the queries ask for the state of the *i*th-bit, i.e., Q executes *bit query*. O’s lies occur at *random*, are *independent* and *context-dependent*. Specifically, O lies with probability $e_0$ and $e_1$ in case of the truthful answer being ”No” and “Yes”, respectively. The particular cases $e_0=0$ and $e_1=0$ have been studied in the context of *half-lies* [24].

The majority of studies on the Rényi-Ulam game to date is *combinatorial*. That is, they stipulate the number of lies (or half-lies) being *a priori* known and focus on finding optimal search strategies which uses a minimum number of queries to identify the target in all cases [23, 24].

Within the framework of this paper, we study the manifestation of the oracle, which is embodied as an indicator random variable whose distribution is derived from a modularity analysis on RNA MFE-structures, see “Path identification”. In the manifestation, erroneous responses arise intrinsically at random: either as a result of the distribution of the random variable (r.v.) or intrinsic errors of the experimental data.

By construction, this rules out a unique winning strategy for Q: instead, we consider the *average fidelity* or accuracy to identify the target utilizing a *sub-optimal* number of queries. We shall propose an entropy-based strategy: at any point a query is selected relative to the subset of bit strings coinciding with the target in all previously identified positions, that maximizes the uncertainty reduction on the subset.

### The Boltzmann ensemble

At a given point in time, an RNA sequence, ${\mathbf {x}}$, assumes a fixed secondary structure, by establishing base pairings. Over time, however, ${\mathbf {x}}$ assumes a plethora of RNA secondary structures appearing at specific rates, see Appendix A for details and context on RNA. These exist in an equilibrium ensemble expressed by the partition function [3] of ${\mathbf {x}}$.

More formally, the *structure ensemble*, $\Omega$ of ${\mathbf {x}}$ is a discrete probability space over the set of all secondary structures, equipped with the probability *p*(*s*) of ${\mathbf {x}}$ folding into *s*. We shall assume that the ensemble of structures is in thermodynamic equilibrium, the distribution of these structures being described as a Boltzmann distribution. The *Boltzmann probability*, *p*(*s*), of the structure *s* is a function of the *free energyE*(*s*) of the sequence ${\mathbf {x}}$ folding into *s*, computed via the Turner energy model [25, 26], see Appendix B for details. The Boltzmann probability *p*(*s*) is expressed as the Boltzmann factor $\exp {(-E(s)/R T)}$, normalized by the *partition function*, $Z=\sum _{s\in \Omega } \exp {(-E(s)/R T)}$, i.e.

$$\begin{aligned} p(s) = \frac{\exp {(-E(s)/R T)}}{Z}, \end{aligned}$$

where *R* denotes the universal gas constant and *T* is the absolute temperature. The Boltzmann distribution facilitates the computation of the partition function *Z* for each substructure. The partition function algorithm [3] for secondary structures computes *Z* and, in particular, the base pairing probabilities based on the free energies for each structure within the structure ensemble $\Omega$.

Let $p_{i,j}$ denote the probability of a base pairing between nucleotides *i* and *j* in the ensemble $\Omega$. Clearly, $p_{i,j}$ can be computed as the sum of probabilities of all secondary structures that contain (*i*, *j*), that is,

$$\begin{aligned} p_{i,j} = \sum _{s\in \Omega } p(s) \delta _{i,j}(s), \end{aligned}$$

where $\delta _{i,j}(s)$ denotes the occurrence of the base pair (*i*, *j*) in *s*.

The thermodynamics-based partition function has been extended to incorporate chemical probing data to generate a Boltzmann ensemble, $\Omega _{\text {probe}}$. These approaches [7, 10, 11] transform structure probing data into a pseudo energy term, $\Delta G(s)$, which reflects how well the structure agrees with the probing data. The Turner free energy is then evaluated by adding the pseudo energy term to the loop-based energy, i.e., $E_{\text {probe}}(s)= E(s)+ \Delta G(s)$. The corresponding equilibrium ensemble, $\Omega _{\text {probe}}$, is distorted in favor of structures that are consistent with probing data, see Appendix C.

For sequences whose probing data are not available, we utilize the 0-1 signature of the target, which is suited for probing data, and quantify the discrepancy between the Boltzmann ensemble and the target via the signature distance $d_{sn}$. The *0-1 signature* of a structure *s* is a 0-1 vector with *k*-th tuple being 0 when the *k*-th base is unpaired in *s*, and 1 otherwise. The *signature distance*$d_{\text {sn}}(s,s')$ between two structures *s* and $s'$ is the Hamming distance between their corresponding 0-1 signatures, see Appendix A. We present that the average distance for an unrestricted ensemble $\Omega$ to a random target is 0.21*n*, while the distance for an ensemble $\Omega _{\text {probe}}$ incorporating simulated probing data is reduced to 0.03*n*, see Appendix D. This motivates us to define a *q*-*Boltzmann ensemble*, $\Omega ^q$, which consists of structures having signature distance to the target *s* at most *qn*, i.e., $\Omega ^q=\{ s'| d_{\text {sn}}(s',s) \le q n \}$. By construction, the computation of $\Omega ^q$ requires the 0-1 signature of the target, and does not need experimental probing data. In particular, we present that the ensemble $\Omega _{\text {probe}}$ has an average normalized signature distance similar to a *q*-ensemble having $q=0.05$. In this paper we discuss unrestricted and restricted Boltzmann ensembles, $\Omega$ and $\Omega ^q$.

**Fig. 3:**
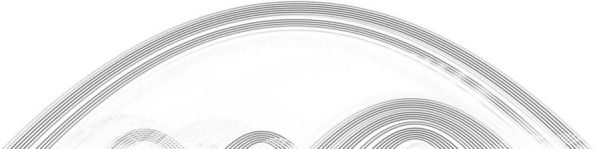
The greyscale diagram of of 1024 Boltzmann sampled structures of a random RNA sequence via ViennaRNA [12]

We shall employ *greyscale diagrams* in order to visualize a sample of secondary structures by superimposing them in one diagram, visualizing the base pairing probabilities. A greyscale diagram displays each base pair (*i*, *j*) as an arc with greyscale $1-p_{i,j}$, where greyscale 0 represents black and 1 represents white, see Fig. 3.

Instead of computing the entire ensemble, we shall consider sub-samples $\Omega '$ consisting of *N* secondary structures with multiplicities of ${\mathbf {x}}$ and refer to $\Omega '$ as the *sample*. For sufficiently large *N* (typically of around size 1000, see [4]), $\Omega '$ provides a good approximation of the Boltzmann ensemble $\Omega$.

A sample $\Omega '$ is a multiset of cardinality *N* and for each structure *s* in $\Omega '$, its *multiplicity*, *f*(*s*), counts the frequency of *s* appearing in $\Omega '$. Thus in the context of $\Omega '$, *p*(*s*) is given by the *s*-multiplicity divided by *N*, $p(s)=f(s)/N$. The base pairing probability $p_{i,j}$ has its $\Omega '$-analogue *f*(*i*, *j*)/*N*, where *f*(*i*, *j*) denotes the frequency of the base pair (*i*, *j*) appearing in $\Omega '$. We shall develop our framework in the context of the structure ensemble $\Omega$, and only reference the sample $\Omega '$, in case the results are particular to $\Omega '$.

### The bit queries

Any structure over *n* nucleotides is considered as a bit string of dimension $$\left( {\begin{array}{c}n\\ 2\end{array}}\right) $$, stipulating (1) a structure is completely determined by the set of base pairs it contains and (2) any position can pair with any other position, except of itself.

The bit query now determines a single bit, i.e. whether or not the base pair (*i*, *j*) is present in the target, stipulating that a unique target is assumed by the sequence in question. We associate the query about the target with a random variable, $X_{i,j}$, defined on the ensemble, via questioning the presence of (*i*, *j*) in each structure. By construction, the distribution of $X_{i,j}$ is given by the base pairing probability ${\mathbb {P}}(X_{i,j}(s)=1)=p_{i,j}$.

Any base pair, (*i*, *j*), has an *entropy*, defined by the information entropy of $X_{i,j}$, i.e.

$$\begin{aligned} H(X_{i,j})=-p_{i,j} \log _2 p_{i,j} -(1-p_{i,j} ) \log _2 (1-p_{i,j} ), \end{aligned}$$

where the units of *H* are in bits. The entropy $$H(X_{i,j}) $$ measures the uncertainty of the base pair (*i*, *j*) in $$\Omega $$ . When a base pair (*i*, *j*) is certain to either exist or not, its entropy $$H(X_{i,j})$$ is 0. However, in case $$p_{i,j} $$ is closer to 1/2, $$H(X_{i,j})$$ becomes larger.

The r.v. $X_{i,j}$ partitions the space $\Omega$ into two disjoint sub-spaces $\Omega _0$ and $\Omega _1$, where $\Omega _k=\{s\in \Omega :X_{i,j}(s)=k\}$ ($k=0,1$), and the induced distributions are given by

$$\begin{aligned} p_0(s)=\frac{p(s)}{1-p_{i,j}} \quad \text { for } s\in \Omega _0,\qquad p_1(s)=\frac{p(s)}{p_{i,j}} \quad \text { for } s\in \Omega _1. \end{aligned}$$

Intuitively, $$H(X_{i,j}) $$ quantifies the average bits of information we would expect to gain about the ensemble when querying a base pair (*i*, *j*). This motivates us to consider the *maximum entropy base pairs*, the base pair $$(i_0,j_0)$$ having maximum entropy among all base pairs in $$\Omega $$, i.e.

$$\begin{aligned} (i_0,j_0)= \mathop {\mathrm{argmax}}\limits _{(i,j)} H(X_{i,j}). \end{aligned}$$

As we shall prove in “Entropy” section, $X_{i_0,j_0}$ produces maximally balanced splits.

### The ensemble tree

Equipped with the notion of ensemble and bit query (i.e. the respective maximum entropy base pairs), we proceed by describing our strategy to identify the target structure as specified in Problem 1. The first step consists in having a closer look at the space of ensemble reductions.

Each split obtained by partitioning the ensemble $\Omega$ using r.v. $X_{i,j}$, can in turn be bipartitioned itself via any of its maximum entropy base pairs. This recursive splitting induces the *ensemble tree*, $T(\Omega )$, whose vertices are sub-samples and in which its *k*-th layer represents a partition of the original ensemble into $2^k$ blocks. $T(\Omega )$, is a rooted binary tree, in which each branch represents a $X_{i,j}$-induced split of the parent into its two children.

Formally the process halts if either the resulting sub-spaces are all *homogeneous*, i.e. their structural entropy is 0, which means that they contain only copies of one structure, or it reaches a predefined maximum level *L*. In our case we set the maximum level to be $L=\log _2 N+1=11$, that is, the height of the ensemble tree is at most 10. The procedure is described as follows:

1. start with the ensemble $\Omega$.
2. for each space $\Omega _{{\mathbf {k}}}$ with $H(\Omega _{{\mathbf {k}}})>0$, where ${\mathbf {k}}$ is a sequence of 0s and 1s having length at most $L-1=10$, compute:
  select the maximum entropy base pair $X_{i_{{\mathbf {k}}},j_{{\mathbf {k}}}}$ of $\Omega _{{\mathbf {k}}}$ as the feature, i.e.
    $$\begin{aligned} X_{i_{{\mathbf {k}}},j_{{\mathbf {k}}}}= \mathop {\mathrm{argmax}}\limits _{(i,j) \text { in } \Omega _{{\mathbf {k}}}} H(X_{i,j}). \end{aligned}$$
  split $\Omega _{{\mathbf {k}}}$ into sub-spaces $\Omega _{{\mathbf {k}}0}$ and $\Omega _{{\mathbf {k}}1}$ using the feature $X_{i_{{\mathbf {k}}},j_{{\mathbf {k}}}}$, that is, $\Omega _{{\mathbf {k}}l }=\{s\in \Omega _{{\mathbf {k}}} :X_{i_{{\mathbf {k}}},j_{{\mathbf {k}}}}(s)=l\}$ for $l=0,1$,
3. repeat Step 2 until all new sub-spaces either have structural entropy 0 or reach the maximum level 11.

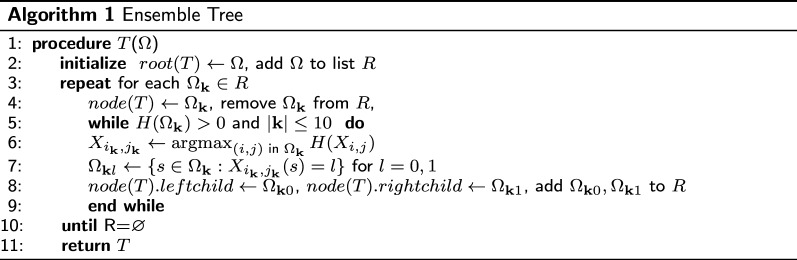

**Fig. 4:**
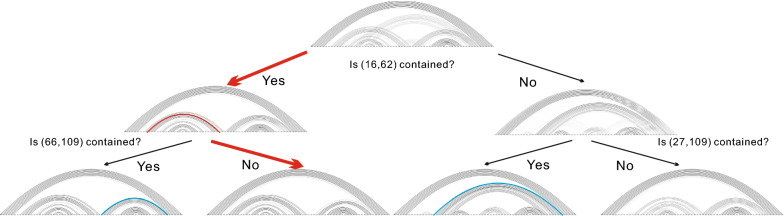
An ensemble tree having maximum level 3. A path from the root to a leaf is identified in color red

In Fig. 4 we display an ensemble tree. We would remark that the ensemble tree may not be complete. The reason is that, when a sub-sample has entropy 0 and thus consists of only one structure, the splitting of this sub-sample will stop.

Clearly, for each space $\Omega _{{\mathbf {k}}}$, the entropies of base pairs can be computed via the traversal of each bit in each structure, and the number of bit queries grows quadratically in the sequence length *n*. Thus finding the maximum entropy base pair can be implemented in quadratic time $O(|\Omega _{{\mathbf {k}}}|\cdot n^2)$, with respect to the sequence length *n*. Since the sum of sizes of spaces on level *i* equals to *N*, each level of the ensemble tree requires $O(N\cdot n^2)$ computations. Therefore the time complexity of Algorithm 1 is $O(L\cdot N\cdot n^2)$, i.e., quadratic with respect to the sequence length *n*, assuming the maximum level *L* and the ensemble size *N* are constants. Via indexing structures in $\Omega$ and memorizing indices in $\Omega _{{\mathbf {k}}}$, Algorithm 1 requires *O*(*N*) memory on each level, i.e., its space complexity is $O(L\cdot N)$.

## Path identification

Given the ensemble tree, we shall construct a path recursively starting from the root to identify the leaf that contains the target. We shall do so by successive bit queries about maximum entropy base pairs, see Fig. 4.

As mentioned before, we employ two manifestations of the oracle, one using modularity based on RNA-folding, and the other determining the existence of base pairs by other experimental means.

### The oracle via modularity and RNA folding

Here we shall employ *modularity* of RNA structures, i.e.  the loops, which constitute the additive building blocks for the free energy have only marginal dependencies. This can intuitively be understood by observing that any two loops can only intersect in at most two nucleotides, see Appendix B.

Let us introduce the notion of embedding and extraction of a contiguous subsequence or fragment, which are functions $\epsilon _{i,j} :B^{n-m} \times B^{m} \rightarrow B^{n}$ and $\xi _{i,j} :B^{n} \rightarrow B^{n-m} \times B^{m}$ given by

$$\begin{aligned} \epsilon _{i,j}((x_i,\dots , x_j),(y_1,\dots ,y_m))&= (y_1,\dots ,y_{i-1},x_i,\dots ,x_j,y_{i+1},\dots y_m)\\ \xi _{i,j}(x_1,\dots ,x_{n})&= ((x_i,\dots ,x_{j}),(x_1,\dots ,x_{i-1},x_{j+1},\dots ,x_{n})). \end{aligned}$$

where *B* denote the set of bases $\{\mathbf{A,U,C,G } \}$, and $j-i+1=n-m$. By construction, we have $\epsilon _{i,j}\circ \xi _{i,j}=\text {id}$ and a contiguous subsequence or fragment of an RNA sequence is called *modular* if it being extracted folds into the same arc configuration as it does embedded in the sequence.

Next we show how to employ probing data to reliably answer whether or not a particular (maximum entropy) arc is contained in the target structure. Structural modularity implies that if this arc can indeed be found in the target structure, then a comparative analysis of the probing data of the entire sequence with those of the extracted sequence, as well as the remainder, concatenated at the cut points will exhibit distinctive similarity. Modularity is a decisive discriminant, if, in contrast, random fragments do not exhibit such similarity.

To quantify to what extent modularity can discriminate base pairs, we perform computational experiments on random sequences via splittings. For each sequence, we consider its MFE structure *s* computed via ViennaRNA [12]. We shall utilize the 0-1 signature of the MFE to mimics its probing data. Given two positions *i* and *j*, we cut the entire sequence ${\mathbf {x}}$ into two fragments, ${\mathbf {x}}_{i,j}$ and the remainder $\bar{{\mathbf {x}}}_{i,j}$, i.e., $\xi _{i,j}({\mathbf {x}})=({\mathbf {x}}_{i,j},\bar{{\mathbf {x}}}_{i,j})$. Subsequently, the two fragments ${\mathbf {x}}_{i,j}$ and $\bar{{\mathbf {x}}}_{i,j}$ refold into their MFE structures $s_{i,j}$ and ${\bar{s}}_{i,j}$, respectively, which are combined into a structure $\epsilon _{i,j}(s_{i,j}, {\bar{s}}_{i,j})$. If bases *i* and *j* are paired in *s*, such a splitting is referred to as *modular* and the resulting structure is denoted by $s'$. Otherwise, it is called *random*, with the output structure $s''$. We proceed by computing the base-pair and signature distance from the MFE *s* to the structures $s'$ or $s''$. The base-pair distance is one of the most frequently used metrics to quantify the similarity of two different structures viewed as bit strings [27, 28], the signature distance measures the similarity between their signatures, which is well suited within the context of the probing profiles, see Appendices A and D.

In the above computation, we run through all possible positions *i* and *j*. For fragments ${\mathbf {x}}_{i,j}$ and $\bar{{\mathbf {x}}}_{i,j}$, we compute the corresponding MFEs and distances. Accordingly, the time complexity of the computation is $O(n^6)$, given by $O(n^2)$ choices of indices times the $O(n^3)$ complexity of the MFE folding and the linear time of the distance computation, where *n* is the sequence length.

**Fig. 5:**
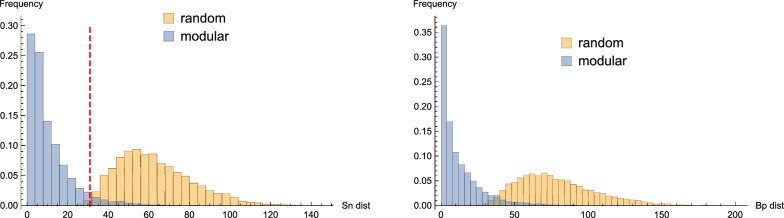
The distributions of the signature distances (LHS) $d_{\text {sn}}(s,s'),d_{\text {sn}}(s,s'')$ and the base-pair distances (RHS) $d_{\text {bp}}(s,s'),d_{\text {bp}}(s,s'')$ obtained from modular splitting (blue) and random splitting (orange). We generated 8000 random sequences ${\mathbf {x}}$ of length 500, and computed their structures *s* (MFE). For any two positions *i* and *j*, we compute $s'$ (modular), $s''$ (random) via ViennaRNA [12]. The red dashed line (left) denotes “threshold distance”, 31 (see main text)

Figure 5 (LHS) compares the distribution of the signature distances $d_{\text {sn}}(s,s')$ and $d_{\text {sn}}(s,s'')$ obtained from modular and random splittings, respectively. The structures induced by modular splitting have much more similar signatures to their MFE structures, than those induced by random splitting. The situation is analogous for base-pair distances, see Fig. 5 (RHS). Since these distances measure structural similarity, the data also indicates that, when *i* and *j* form a base pair in *s*, the fragment ${\mathbf {x}}_{i,j}$ is more likely to fold into the same configuration as it does being embedded, i.e. ${\mathbf {x}}_{i,j}$ is modular.

**Fig. 6:**
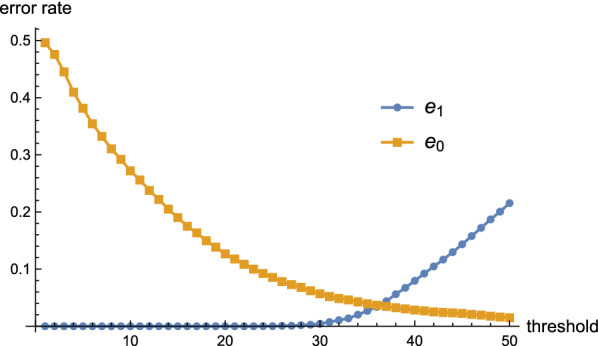
The error rates $e_0$ and $e_1$ as a function of the threshold $\theta$. We use the same sequences and structures as described in Fig. 5. When the signature distance is smaller than $\theta$, we predict that bases *i* and *j* are paired, otherwise they are unpaired. The error rates $e_0$ and $e_1$ are given by the false omission rate (FOR) and false discovery rate (FDR), respectively

The data displayed in Fig. 5 suggests the threshold distance, $\theta$, for signatures, by which we distinguish modular from random. More specifically, if the signature distance is smaller than $\theta$, we predict that bases *i* and *j* are paired. Otherwise they are unpaired. In order to quantify the accuracy of this classification, we consider the resulting false discovery rate (FDR) and false omission rate (FOR).^1^ In our Rényi-Ulam game variation, the expected values of FDR and FOR are the error rates $e_1$ and $e_0$ in case the truthful answer being yes and no, respectively. Figure 6 displays the error rates $e_0$ and $e_1$ as functions of $\theta$. For $\theta =31$, we compute $e_0\approx 0.052$ and $e_1 \approx 0.007$, i.e.  we have an error rate of 0.052 for rejecting and an error rate of 0.007 for confirming a base pair.

### A new fragmentation

Equipped with the ensemble tree and the oracle via modularity, our framework provides a fragmentation process combining “local” probing profiles with the “global” one via modularity. The novel fragmentation process is guided by the base-pair queries of the ensemble tree inferred from the restricted Boltzmann sample incorporating chemical probing. Given the maximum entropy base pair, (*i*, *j*), extraction splits the sequence into two fragments, one being the extracted fragment ${\mathbf {x}}_{i,j}$ and the other, $\bar{{\mathbf {x}}}_{i,j}$. We perform probing experiments on these two segments, and obtain the reactive probabilities ${\mathbf {q}}_{i,j}$ and $\bar{{\mathbf {q}}}_{i,j}$, respectively. Let ${\mathbf {q}}$ be the reactive probability for the entire sequence, and ${\mathbf {q}}'$ be the embedding of ${\mathbf {q}}_{i,j}$ into $\bar{{\mathbf {q}}}_{i,j}$, i.e., ${\mathbf {q}}'= \epsilon _{i,j} ({\mathbf {q}}_{i,j}, \bar{{\mathbf {q}}}_{i,j})$. As shown in the previous subsection, if the Hamming distance $d({\mathbf {q}},{\mathbf {q}}')$ is smaller than threshold $\theta$, then the probing profiles are similar, i.e., two bases *i* and *j* are paired. Otherwise, they are unpaired in the target structure.

The fragmentation procedure can be summarized as follows:

1. a probing experiment for the entire sequence is performed and the reactive probability ${\mathbf {q}}$ is obtained,
2. a Boltzmann sample $\Omega _{\text {probe}}$ of *N* structures, consistent with the probing data ${\mathbf {q}}$ is computed,
3. the ensemble tree $T(\Omega )$ containing the sub-spaces $\Omega _{{\mathbf {k}}}$ and the corresponding maximum entropy base pairs $X_{i_{{\mathbf {k}}},j_{{\mathbf {k}}}}$ is constructed,
4. starting with $\Omega$ we recursively answer the queries, determining thereby a path through the ensemble tree from the root to a leaf.
5. once in a leaf, Proposition 1 guarantees the existence of a distinctive structure which we stipulate to be the target structure.

**Fig. 7:**
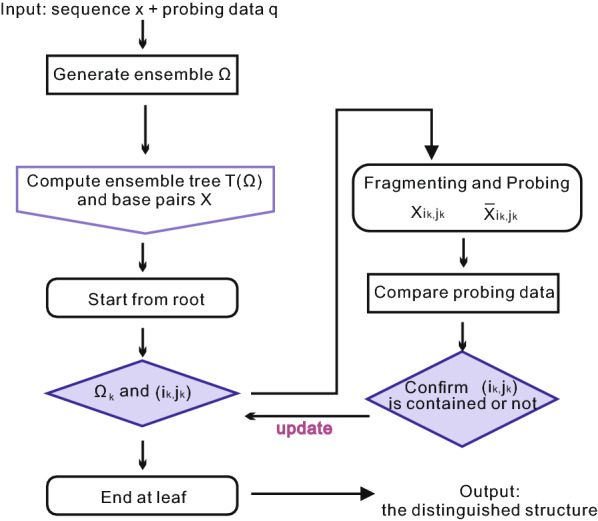
The workflow diagram of our fragmentation process

Figure 7 demonstrates the workflow of the fragmentation process, which can be considered as an implementation of our overall strategy in Fig. 2 (LHS), via incorporating the new fragmentation process into the workflow.

We would point out that the key of path identification is determination of base pairs. Instead of using modularity and “local” probing data, we can also apply other experimental approaches to identifying base pairs. In Appendix E, we summarize state-of-the-art experimental approaches that could possibly be utilized to determine base pairs and to identify the path in the ensemble tree. In particular, we detail two methods, both of which utilize chemical probing data in different ways than our fragmentation [29, 30] and recover base pairs with a false discovery rate less than 0.05.

## Results

Given an input sample $\Omega$, we construct the ensemble tree $T(\Omega )$ having maximum level $L=11$, recursively computing the maximum entropy base pairs as described in Algorithm 1. In this section, we shall analyze the entropy of leaves in order to quantify the existence of a distinguished structure and to identify the target.

### Entropy

To quantify the uncertainty of an ensemble, we define the *structural entropy* of an ensemble, $\Omega$, of an RNA sequence, ${\mathbf {x}}$, as the Shannon entropy

$$\begin{aligned} H(\Omega ) = -\sum _{s\in \Omega } p(s) \log _2 p(s), \end{aligned}$$

the units of *H* being bits. The sum is taken over all secondary structures *s* of ${\mathbf {x}}$, and *p*(*s*) denotes the Boltzmann probability of the structure *s* in the ensemble $\Omega$. The notion of structural entropy is originated in thermodynamics and is usually regarded as a measure of disorder, or randomness of an ensemble [31, 32].

Given a sample $\Omega '$ of size *N*, the structural entropy has the upper bound $\log _2 N$, that is, $H_{}(\Omega ')$ reaches its maximum when all sampled structures are different. Throughout the paper, we assume $N=1024$ and therefore $H_{}(\Omega ')\le 10$.

#### **Proposition 1**

*Let*$\Omega '$* be a sample having structural entropyE*, *where*$0\le E \le 1$. *Then there exists one structure in*$\Omega '$*having probability at leastf*(*E*), *wheref*(*E*) *is the solution of the equation*

$$ -p \log _2 p -(1-p) \log _2 (1-p)=E $$

**Fig. 8:**
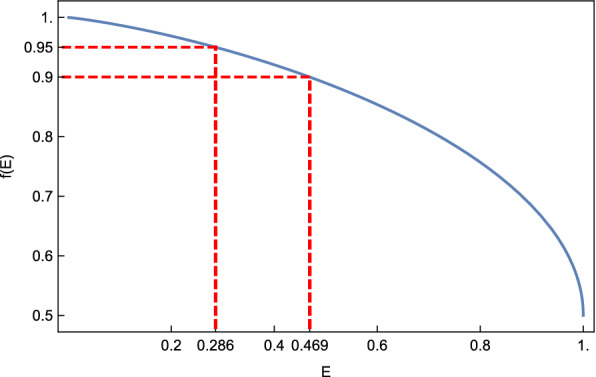
A sample with structural entropy *E* contains a distinguished structure having probability at least *f*(*E*)

*satisfying*$0.5\le p \le 1$. *In particular, we have*$f(1)=0.5$, $f(0.469) \approx 0.9$*and*$f(0.286) \approx 0.95$, *see Fig.* 8.

Proposition 1 implies that a sample with small structural entropy contains a distinguished structure and a proof is given in Appendix F. We refer to a sample having a distinguished structure of probability at least $\lambda$ as being $\lambda$-*distinguished*.

Next we quantify the reduction of a bit query on an ensemble. Recall that the associated r.v. $X_{i,j}$ of a base pair (*i*, *j*) partitions the sample $\Omega$ into two disjoint sub-samples $\Omega _0$ and $\Omega _1$, where $\Omega _k=\{s\in \Omega :X_{i,j}(s)=k\}$ ($k=0,1$).

The *conditional entropy*, $H(\Omega |X_{i,j})$, represents the expected value of the entropies of the conditional distributions on $\Omega$, averaged over the conditioning r.v. $X_{i,j}$ and can be computed by

$$\begin{aligned} H(\Omega |X_{i,j})= (1-p_{i,j}) H(\Omega _0)+ p_{i,j} H(\Omega _1). \end{aligned}$$

Then the *entropy reduction*$R(\Omega ,X_{i,j})$ of $X_{i,j}$ on $\Omega$ is the difference between the *a priori* Shannon entropy $H(\Omega )$ and the conditional entropy $H(\Omega |X_{i,j})$, i.e.

$$\begin{aligned} R(\Omega ,X_{i,j})= H(\Omega )-H(\Omega |X_{i,j}). \end{aligned}$$

The entropy reduction quantifies the average change in information entropy from an ensemble in which we cannot tell whether or not a certain structure contains (*i*, *j*), to its bipartition where one of its two blocks consists of structures that contain (*i*, *j*) and the other being its complement.

#### **Proposition 2**

*The entropy reduction* $R(\Omega ,X_{i,j})$ *of* $X_{i,j}$ *is given by the entropy* $H(X_{i,j})$ *of* $X_{i,j}$ *, i.e.*

2 $$\begin{aligned} R(\Omega ,X_{i,j})= H(X_{i,j}). \end{aligned}$$

Proposition 2 queries a Bernoulli random variable inducing a split, reducing its average conditional entropy exactly by the entropy of the random variable itself. In the context of the Rényi-Ulam game, Q asks a question that helps to maximally reduce the space of possibilities. A proof of Proposition 2 is presented in Appendix G.

The next observation shows that querying maximum entropy base pairs, induces a best possible balanced split of the ensemble.

#### **Proposition 3**

*Suppose that*$X_{i,j}$*induces a partition of the ensemble*$\Omega$*into sub-samples*$\Omega _0^{i,j}$*and*$\Omega _1^{i,j}$. *Let*$(i_0,j_0)$*be a maximum entropy base pair of*$\Omega$. *Then we have*

1. $(i_0,j_0)$*minimizes the difference of the probabilities of the two sub-samples,*
  $$\begin{aligned} |{\mathbb {P}}(\Omega _0^{i_0,j_0}) -{\mathbb {P}}(\Omega _1^{i_0,j_0})|\le |{\mathbb {P}}(\Omega _0^{i,j}) -{\mathbb {P}}(\Omega _1^{i,j})|, \end{aligned}$$
  *for any (i*, *j). Here we define*${\mathbb {P}}(\Omega _k^{i,j}) = {\mathbb {P}}(s\in \Omega :X_{i,j}(s)=k)$*with*$k=0,1$.
2. $(i_0,j_0)$*maximizes the entropy reduction*$R(\Omega ,X_{i,j})$*of*$X_{i,j}$*on*$\Omega$,
  $$\begin{aligned} R(\Omega ,X_{i_0,j_0}) \ge R(\Omega ,X_{i,j}), \end{aligned}$$
  *for any (i*, *j)*.

Proposition 3 first shows that the bit query about the maximum entropy base pair $X_{i_0,j_0}$ partitions the ensemble as balanced as possible, i.e. into sub-samples having the minimum difference of their probabilities. It furthermore establishes that the splits have minimum average structural entropy (or uncertainty), since $X_{i_0,j_0}$ provides the maximum entropy reduction on the ensemble. Thus the query about $(i_0,j_0)$ is the most informative among all bit queries.

Finally we quantify the average entropy of sub-samples, $\Omega _{t}$, on the *t*-th level of the ensemble tree, and establish the existence of a distinguished structure. The analysis of entropies depends of course on the way the samples are being constructed. To this end, given a random sequence, we construct the ensemble tree for two types of samples, one being unrestricted samples of structures, $\Omega$, and the other utilizing *q*-Boltzmann sampling that incorporates the signature of the target, $\Omega ^q$, see “The Boltzmann ensemble” section. Specifically, the target structure is randomly selected from the unrestricted sample, and the *q*-Boltzmann sample utilizes the 0-1 signature of the target. We would point out that our framework does not require to ”choose” the target for a sequence, and here we make the choices to facilitate the computation on random sequences.

**Fig. 9:**
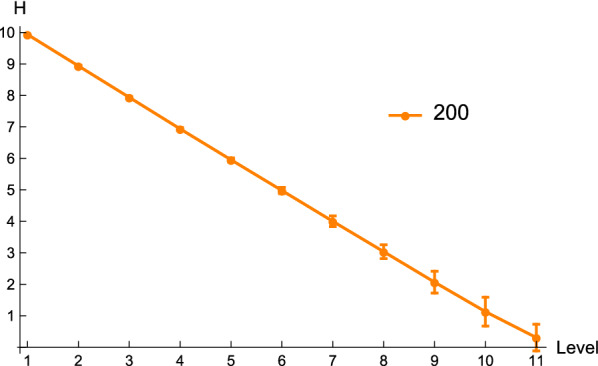
The average entropy of sub-samples $H(\Omega _{t})$ on the *t*-th level. We randomly generate 1000 sequences of length 200, and sample $2^{10}$ structures together with a target structure *s* for each sequence

For unrestricted Boltzmann samples, the structural entropy $H(\Omega _{t})$ of sub-samples on the *t*-th level decreases, as the level *t* increases, see Fig. 9. In particular, the average entropy $H(\Omega _{11})$ of leaf samples is 0.328 and 0.147, for sequences having 200 and 300 nucleotides, respectively. Proposition 1 guarantees that the leaf $\Omega _{11}$ is 0.90-distinguished, i.e. containing a distinguished structure with ratio at least 0.90 for sequences of length 200, and 0.95-distinguished for sequences of length 300.

**Fig. 10:**
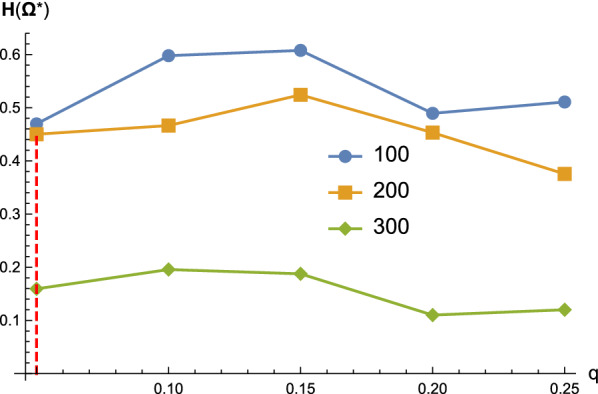
The structural entropy $H(\Omega ^q_{11})$ of the leaf sub-samples for different *q*-values. We randomly generate 1000 sequences of length 100, 200 and 300. For each sequence, we then generate a *q*-Boltzmann sample $\Omega ^q$ of $2^{10}$ structures together with a target *s*. The red dashed line denotes *q*-samples having $q=0.05$, which is tantamount to Boltzmann samples $\Omega _{\text {probe}}$ incorporating the probing data via pseudo-energies

For *q*-Boltzmann samples $\Omega ^q$ of structures having signature distance to the target *s* at most *qn*, the small entropy of the leaf and the high ratio of the distinguished structure are robust over a range of *q*-values, see Fig. 10. We also observe that, for longer sequences, the entropy is smaller, and therefore the ratio of the distinguished structure is higher.

### Target identification

Any leaf of the ensemble tree exhibiting a structural entropy less than one, contains, by Proposition 1, a distinguished structure. Successive queries produce a unique, distinguished leaf, $\Omega ^*$ which, with high probability, contains structures that are compatible with the queries. Let $s^*$ be the distinguished structure in $\Omega ^*$, and *s* denote the target.

In this section, we shall analyze this probability, ${\mathbb {P}}(s\in \Omega ^*)$, as well as ${\mathbb {P}}(s^*=s)$ and ${\mathbb {P}}(s^*=s\mid s\in \Omega ^*)$, see Table 1. For the path identification to the leaf $\Omega ^*$, we consider the error rates $e_0=0.05$ and $e_1=0.01$ computed in “Path identification” section.

**Fig. 11:**
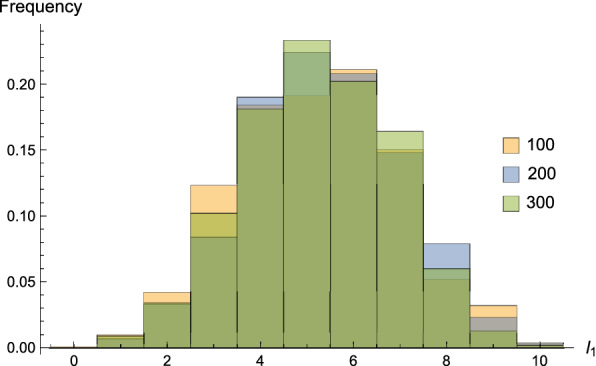
The distributions of $l_1$, the number of queried base pairs on the path that are confirmed by the target structure. We generate unrestricted Boltzmann samples for random sequences of different lengths

**Fig. 12:**
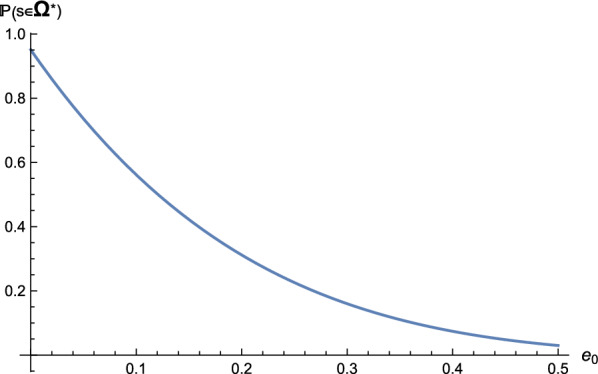
The probability ${\mathbb {P}}(s\in \Omega ^* )$ as a function of the error rate $e_0$, for fixed $e_1=0.01$ and $l_0=l_1=5$

As detailed in “Path identification” section, these probabilities depend on the error rates $e_0$ and $e_1$, and since these errors occur independently, we derive ${\mathbb {P}}(s\in \Omega ^* )=(1-e_0)^{l_0} (1-e_1)^{l_1}$, where $l_0$ and $l_1$ denote the number of No-/Yes-answers to queried base pairs along the path, respectively. Figure 11 displays the distribution of $l_1$. We observe that $l_1$ has a mean around 5, i.e., the probabilities of queried base pairs being confirmed and being rejected are roughly equal. For $l_0=l_1=5$, we have a theoretical estimate ${\mathbb {P}}(s\in \Omega ^* )\approx 0.736$. In Fig. 12 we present that ${\mathbb {P}}(s\in \Omega ^* )$ decreases as the error rate $e_0$ increases, for fixed $e_1=0.01$.

For (unrestricted) Boltzmann samples generated from random sequences, we present the probability ${\mathbb {P}}(s\in \Omega ^*)$ of the leaf containing the target is greater than $74\%$, which agrees with the above theoretical estimate. Note that this amounts to having no probing data as a constraint for the sampled structures, a worst case scenario, so to speak.

**Table 1:** Key observables

Quantity	Description
${\mathbb {P}}(s\in \Omega ^*)$	The probability of the target being in the leaf
${\mathbb {P}}(s^*=s)$	The probability of the distinguished structure being identical to the target
${\mathbb {P}}(s^*=s\mid s\in \Omega ^*)$	The probability of correctly identifying the target, given that it is in the leaf

Furthermore, the probability that the distinguished structure is identical to the target is approximately unchanged, see Table 2. ${\mathbb {P}}(s^*=s\mid s\in \Omega ^*)$ indicates, that once we are in the correct leaf, the chance of correctly identifying the target increases to $94\%$ for sequences of length 300. Accordingly, the key factor is the correct identification of the leaf $\Omega ^*$.

**Table 2:** Target identification: we randomly generate 1000 sequences of length *n* and Boltzmann sample $2^{10}$ structures together with a target structure *s* for each sequence

	$n=100$	$n=200$	$n=300$
${\mathbb {P}}(s\in \Omega ^*)$	$0.768 \pm 0.178$	$0.742 \pm 0.192$	$0.751 \pm 0.187$
${\mathbb {P}}(s^*=s)$	$0.669 \pm 0.222$	$0.646\pm 0.229$	$0.706 \pm 0.208$
${\mathbb {P}}(s^*=s\mid s\in \Omega ^*)$	$0.871 \pm 0.288$	$0.871 \pm 0.309$	$0.940 \pm 0.277$

We compute the probabilities of identifying the target utilizing the ensemble tree. We display mean and standard deviation

**Fig. 13:**
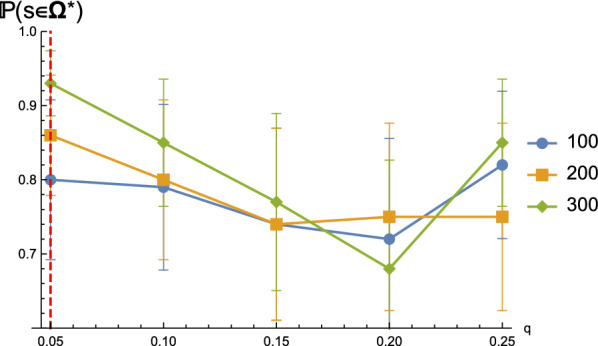
The probability ${\mathbb {P}}(s\in \Omega ^{*} )$ of being in the correct leaf for different *q*-values. We use the same sequences and *q*-Boltzmann samples as described in Fig. 10. Error bars show the standard deviations for random sequence samplings in the corresponding cases. The red dashed line denotes *q*-samples having $q=0.05$, which is tantamount to Boltzmann samples $\Omega _{\text {probe}}$ incorporating the probing data via pseudo-energies

For *q*-Boltzmann samples $\Omega ^q$ filtered by signature distance $\le q n$ we observe the following: the probability ${\mathbb {P}}(s\in \Omega ^{*} )$ of the leaf to contain the target is greater than $70\%$ is robust over a range of *q*-values, see Fig. 13. As expected, as *q* increases, the probability of the target being in the correct leaf decreases, due to the fact that the *q*-samples become less constraint by the probing data.

In particular, we observe that, for $q=0.05$ and sequences of length 300, the probability of the ensemble tree identifying the correct leaf is greater than $90\%$, see Fig. 13 (red dashed line). As the Boltzmann ensembles incorporation of probing data via pseudo-energies result in a *q*-value of 0.05, this translates into ${\mathbb {P}}(s\in \Omega ^{*})\ge 90\%$ for such ensembles generated by such restricted Boltzmann samplers for sequences of length 300.

**Fig. 14:**
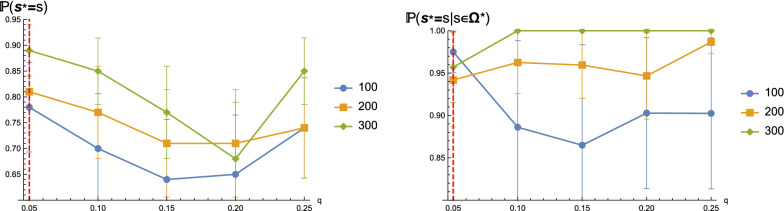
The probabilities ${\mathbb {P}}(s^*=s)$ (LHS) and ${\mathbb {P}}(s^*=s\mid s\in \Omega ^*)$ (RHS) of correctly identifying the target, either in general or conditioning on being in the correct leaf. We use the same sequences and *q*-Boltzmann samples as described in Fig. 10. Error bars show the standard deviations for random sequence samplings in the corresponding cases. The red dashed line denotes *q*-samples having $q=0.05$, which is tantamount to Boltzmann samples $\Omega _{\text {probe}}$ incorporating the probing data via pseudo-energies

We demonstrate that the ensemble tree localizing the target with high fidelity is robust, across samples of sequences having various lengths and different signature filtration *q*. Figure 14 (LHS) shows that the ensemble tree for longer sequences has a higher chance of identifying the target. Once we are in the correct leaf, the chance of correctly distinguishing the target significantly increases, from around $75\%$ to over $94\%$ in the case of sequences having 200 nucleotides, see Fig. 14 (RHS).

As mentioned above, the key is the correct identification of the leaf containing the target, and its distinguished structure to coincide with the latter. These events are quantified via ${\mathbb {P}}(s\in \Omega ^{*} )$ and ${\mathbb {P}}(s^*=s)$, which depend on the error rates $e_0$ and $e_1$.

These error rates can be reduced by asking the same query repeatedly. In our Rényi-Ulam game, repeating the same query is tantamount to performing the same experiment multiple times. It is reasonable to assume that experiments are performed independently and thus errors occur randomly. Intuitively, repeated experiments reduce errors originated from the noisy nature of experimental data. Utilizing Bayesian analysis, we show that, if we get the same answer to the query twice, the error rates would become significantly smaller, for example, $e_0^{[2]} =0.003$ and $e_1^{[2]} =0.00005$, see Appendix H.

In principle, we can reduce the error rates by repeating the same query *k* times. The error rates would approach to 0 as *k* grows to infinity. In this case, ${\mathbb {P}}(s\in \Omega ^{*} ) \approx 1$, i.e. the leaf always contains the target. The fidelity of the distinguished structure ${\mathbb {P}}(s^{*}=s )$ increases from 70 to $94\%$ for sequences of length 300.

### Robustness

A significant advantage of our approach is the robustness of target identification across Boltzmann samples of various sizes and different GC-contents. For sizes, we deliberately change the number *N* of sampled structures ranging from $2^9$ to $2^{11}$. Accordingly, the maximum level *L* of the ensemble tree varies, i.e., it grows at a logarithmic scale $L=\log _2 N+1$. For GC-contents, we utilize sequences with different nucleotide compositions to generate Boltzmann samples. It is believed that medium or low GC-content offers greater transcription efficiency, while high GC-content provides better structural stability [33]. We thus consider GC-rich and AU-rich random sequences. For each variant sample, we compute the distinguished structure $s^*$ in the leaf $\Omega ^*$ via successive *L* queries, and presents the probabilities of the distinguished structure being identical to the target, see Tables 3 and 4.

In Table 3, we demonstrate that the ensemble tree localizing the target with high fidelity is robust, across unrestricted samples of various sizes. Additionally, Table 3 shows that the ensemble tree for longer sequences has a higher chance of identifying the target, see the probabilities displayed in italics. We also observe that the probability of being in the distinguished leaf, ${\mathbb {P}}(s\in \Omega ^*)$, slightly decreases, as the sample size increases. This can be improved by repeating the same query multiple times as shown in “Target identification” section.

Table 4 shows the robustness of target identification across samples of different GC-contents. This indicates that the effectiveness of our approach remains unaffected by RNA sequences with various GC-contents.

**Table 3:** The robustness of target identification across samples of various sizes

	*N*	$n=100$	$n=200$	$n=300$
${\mathbb {P}}(s\in \Omega ^*)$	$2^{9}$	$0.774 \pm 0.175$	$0.782 \pm 0.171$	$0.761 \pm 0.182$
	$2^{10}$	$0.768 \pm 0.178$	$0.742 \pm 0.192$	$0.751 \pm 0.187$
	$2^{11}$	$0.747 \pm 0.189$	$0.711 \pm 0.206$	$0.738 \pm 0.194$
${\mathbb {P}}(s^*=s)$	$2^{9}$	$0.685 \pm 0.216$	$0.698\pm 0.211$	$0.724 \pm 0.200$
	$2^{10}$	$0.669 \pm 0.222$	$0.646\pm 0.229$	$0.706 \pm 0.208$
	$2^{11}$	$0.682 \pm 0.217$	$0.634 \pm 0.237$	$0.695 \pm 0.212$
${\mathbb {P}}(s^*=s\mid s\in \Omega ^*)$	$2^{9}$	*0.885* ± 0.279	*0.892* ± 0.270	*0.951* ± 0.263
	$2^{10}$	*0.871* ± 0.288	*0.871* ± 0.309	*0.940* ± 0.277
	$2^{11}$	*0.913* ± 0.290	*0.864* ± 0.334	*0.942* ± 0.288

We generate 1000 random sequences of length *n*. For each sequence, we then generate (unrestricted) Boltzmann samples of *N* structures together with a target structure *s*. The size *N* of the samples varies from $2^9$ to $2^{11}$, and the maximum level of the ensemble tree is given by $L=\log _2 N+1$. We compute the probabilities of identifying the target utilizing the ensemble tree. We display mean and standard deviation

**Table 4:** The robustness of target identification across samples of different GC-contents

	GC-content	$n=100$	$n=200$	$n=300$
${\mathbb {P}}(s\in \Omega ^*)$	GC-rich	$0.778 \pm 0.172$	$0.732 \pm 0.196$	$0.735 \pm 0.195$
	Uniform	$0.768 \pm 0.178$	$0.742 \pm 0.192$	$0.751 \pm 0.187$
	AU-rich	$0.773 \pm 0.176$	$0.735 \pm 0.195$	$0.749 \pm 0.188$
${\mathbb {P}}(s^*=s)$	GC-rich	$0.720 \pm 0.202$	$0.655 \pm 0.226$	$0.674 \pm 0.220$
	Uniform	$0.669 \pm 0.222$	$0.646\pm 0.229$	$0.706 \pm 0.208$
	AU-rich	$0.677 \pm 0.219$	$0.655 \pm 0.226$	$0.701 \pm 0.210$
${\mathbb {P}}(s^*=s\mid s\in \Omega ^*)$	GC-rich	$0.925 \pm 0.259$	$0.895\pm 0.309$	$0.917 \pm 0.299$
	Uniform	$0.871\pm 0.288$	$0.871 \pm 0.309$	$0.940\pm 0.277$
	AU-rich	$0.876\pm 0.283$	$0.891 \pm 0.308$	$0.936 \pm 0.280$

We generate 1000 random sequences of length *n* with different GC-contents, where GC-rich sequences consist of $30\%$ Gs, $30\%$ Cs, $20\%$ As and $20\%$ Us; Uniform comprise $25\%$ Gs, $25\%$ Cs, $25\%$ As and $25\%$ Us; AU-rich contain $20\%$ Gs, $20\%$ Cs, $30\%$ As and $30\%$ Us. This process can be done by software such as GenRGenS [34]. For each sequence, we then generate (unrestricted) Boltzmann samples of $N=2^{10}$ structures together with a target structure *s*. We compute the probabilities of identifying the target utilizing the ensemble tree. We display mean and standard deviation

### Performance comparison

Here we apply our approach to natural RNAs, and compare the performance with the RNA structure modeling method developed by Hajdin et al. [8]. First, we use the data set of 18 RNAs with published SHAPE profiles and accepted secondary structures [8]. This data set includes tRNAs, ribosomal RNAs, riboswitches, and viruses. RNA lengths vary from 34 to 530 nucleotides, see Table 5. We consider the accepted secondary structure excluding pseudoknots as the target. Specifically, we reduce a pseudoknot by removing the helix having the minimum size in the pseudoknot. Then, for each sequence, we incorporate chemical probing data as pseudo energies [7] and generate a Boltzmann sample $\Omega _{\text {probe}}$ of $2^{10}$ structures, see “The Boltzmann ensemble” section. We compute the ensemble tree for each sample $\Omega _{\text {probe}}$, and identify the distinguished leaf $\Omega ^*$ via successive base-pair queries on the target. For each base-pair query, we consider the error rates $e_0=0.05$ and $e_1=0.01$ computed in “Path identification” section. We output the distinguished structure $s^*$ in the leaf as the predicted structure.

Here we drop the assumption that the target is always in the sample. We consider the *model-agnostic* property of our approach, i.e., whether it guarantees to find the “best” structure in the sample even if the correct target is not contained. Also, we point out that our framework does not require a priori knowledge of the target structure. However, because we are in lack of probing data on fragments to confirm or reject certain base-pairs using modularity, we need to utilize knowledge of the target to answer the queries.

Nevertheless, as a proof of concept, we present the computational results of our approach applying to the data set. To compare with [8], we compute three measures of performance: sensitivities, positive predictive value (PPV), and accuracy. We show that the accuracy of our method is, on average, 5 percentage points higher than that of [8]. Moreover, the improvement on the target identification accuracy is robust, across sequences of different types and various lengths. Although the sample $\Omega _{\text {probe}}$ does not contain the target structure for sequences longer than 76, the results demonstrate that our approach is capable of identifying the “best” structure, which is defined as the one in the sample having the smallest base-pair distance to the target.

**Table 5:** Target identification results for 18 test sequences with SHAPE profiles from [8]

RNA	Length	Hajdin et al. [8]			Our method		
		Sens	PPV	Acc	Sens	PPV	Acc
Pre-Q1 riboswitch, *B. subtilis*	34	100	100	100	100	100	100
Fluoride riboswitch, *P. syringae*	66	93.8	93.8	93.8	100	100	100
Adenine riboswitch, *V. vulnificus*	71	100	100	100	100	100	100
tRNA(asp), *yeast*	75	95.2	95.2	95.2	100	100	100
tRNA(phe), *E. coli*	76	100	84.0	91.7	100	100	100
TPP riboswitch, *E. coli*	79	95.5	87.5	91.4	95.6	100	97.7
SARS corona virus pseudoknot	82	84.6	88.0	86.3	97.4	84.4	90.5
cyclic-di-GMP riboswitch, *V. cholerae*	97	89.3	86.2	87.7	100	96.6	98.3
5S rRNA, *E. coli*	120	85.7	76.9	81.2	100	97.3	98.6
M-Box riboswitch, *B. subtilis*	154	87.5	91.3	89.4	89.7	97.8	93.5
P546 domain, *bI3 group I intron*	155	94.6	96.4	95.5	98.2	100	99.1
Lysine riboswitch, *T. maritima*	174	87.3	88.7	88.0	94.8	100	97.3
Group I intron, *Azoarcus sp.*	214	92.1	95.1	93.6	96.5	96.5	96.5
Hepatitis C virus IRES domain	336	92.3	96.0	94.1	98.0	97.0	97.5
Group II intron, *O. iheyensis*	412	93.2	97.6	95.4	94.4	100	97.1
Group I Intron, *T. thermophila*	425	93.9	91.2	92.5	96.8	92.3	94.5
5′ domain of 23S rRNA, *E. coli*	511	97.2	76.8	86.4	98.7	83.4	90.4
5′ domain of 16S rRNA, *E. coli*	530	93.0	83.6	88.2	97.6	89.7	93.5
Average		93.0	90.4	*91.6*	97.6	96.4	*96.9*

We consider the accepted secondary structure excluding pseudoknots as the target. Our method identifies the distinguished structure from a Boltzmann sample of $2^{10}$ structures via 10 base-pair queries on the target. For each base-pair query, we consider the error rates of accepting or rejecting a base pair, $e_0=0.05$ and $e_1=0.01$. To compare the structure prediction approach in [8] and our method, we present three measures of performance: sensitivities (Sens), the fraction of pairs in the accepted structure that are predicted ($\text {Sens}=\frac{\text {FP}}{\text {TP}+\text {FN}}$); positive predictive value (PPV), the proportion of predicted pairs that are in the accepted structure ($\text {PPV}=\frac{\text {FP}}{\text {TP}+\text {FP}}$); and accuracy (Acc), the harmonic mean of Sens and PPV ($\text {Acc}=\frac{2\cdot \text {Sens} \cdot \text {PPV}}{\text {Sens}+\text {PPV}}$). The average accuracy of both methods is displayed in italics

## Discussion

**Fig. 15:**
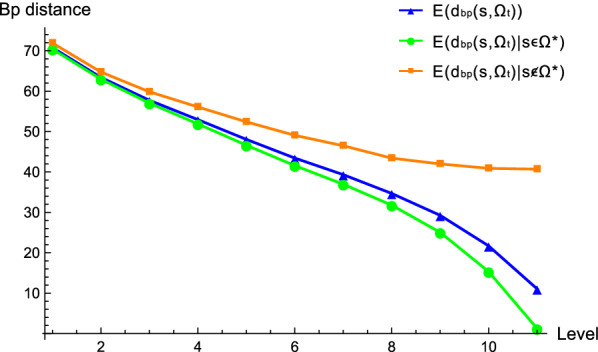
The average base-pair distance $d_{\text {bp}}(s,\Omega _{t})$ between the target *s* and the sub-sample $\Omega _{t}$ on the path. The expectation is taken over all ensemble trees (blue), the set of ensemble trees in which the leaf containing the target is identified (green) and its complement (orange). The computation is based on the Boltzmann samples of sequences of length 300

In our framework, the key factor is the correct identification of the leaf that contains the target. Figure 15 displays the average base-pair distances $d_{\text {bp}}(s,\Omega _{t})$^2^ between the target structure *s* and the *t*-th sub-sample $\Omega _{t}$ on the path. We contrast three scenarios, first the expectation being taken over all ensemble trees (blue), the set of ensemble trees in which the leaf containing the target is identified (green) and its complement (orange). We here present that the correct identification of the leaf containing the target significantly reduces the distance between the target and the sub-samples.

**Fig. 16:**
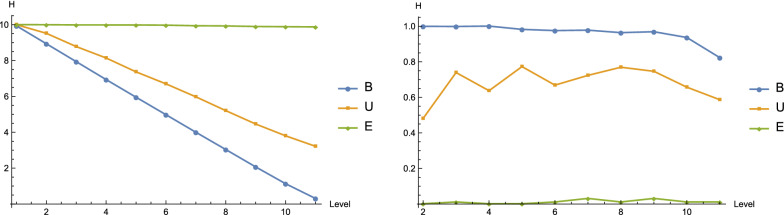
The average entropy of sub-samples $H(\Omega _{t})$ (LHS) and queried base pairs $H(X_{t})$ (RHS) on the *t*-th level of the ensemble tree. We contrast the ensemble trees obtained from a Boltzmann sample (B, blue), a uniform sample (U, orange), or an E-sample (E, green), which is comprised of $2^{10}$ distinct structures, each containing only one base pair. For the former two types of samples, we randomly generate 1000 sequences of length 200. For each sequence, we sample $2^{10}$ structures together with a target structure *s*, according to the Boltzmann or uniform distributions

Our framework is based on two assumptions. The first is sampling from the Boltzmann ensemble of structures. This assumption is important, as for an arbitrary sample, the splittings could be highly unbalanced and the leaf of the ensemble tree does not always contain a distinguished structure. By quantifying the distinguished structure via the flow of entropies of sub-samples on the path, we contrast three classes of samples, the first being a Boltzmann sample (B-sample), the second a uniform sample (U-sample) and the third an E-sample,^3^ see Fig. 16. We present that, in a Boltzmann sample, the entropies of sub-samples on the *t*-th level decrease much more sharply than those in the latter two classes, see Fig. 16 (LHS). In particular, the latter two produce leaves exhibiting an average entropy greater than 1, i.e. not containing a distinguished structure. As proved in Proposition 2, the entropy reduction equals to the entropy of the queried base pair. Figure 16 (RHS) explains the reason for the significant reduction, that is, the maximum entropy base pairs in Boltzmann samples have entropy close to 1 on each level, implying that the bit queries split the ensemble roughly in half each time. The latter two types of samples do not exhibit this phenomenon. In upcoming work, we shall investigate this phenomenon via quantifying how the uncertainty or entropy of the ensemble is distributed in the bit queries.

The second assumption is that the target is contained in the sample. This assumption can be validated by generating samples of larger size, and checking whether or not the distinguished structure is reproducible. We would remark that, even though the probability of identifying the target ${\mathbb {P}}(s^*=s)$ slightly decreases for larger samples (Table 3), we can significantly improve ${\mathbb {P}}(s^*=s)$ by performing the same experiment multiple times, see “Target identification” and Appendix H.

Accordingly, the probability and entropy of a base pair is calculated in the context of the entire ensemble, and thus the ensemble tree together with maximum entropy base pairs. [32] show that the structural entropy of the entire Boltzmann ensemble is asymptotically linear in *n*, i.e. $H(\Omega _{\text {entire}})\approx 0.07 n$. Since each queried base pair reduces the entropy by approximately 1 and the reduction is additive by construction, the ensemble tree would require approximately 0.07*n* queries to identify a leaf that has entropy smaller than 1 and contains a distinguished structure.

**Fig. 17:**
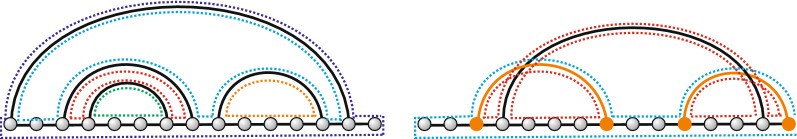
The loop-based decomposition of a secondary structure (LHS) and a pseudoknot (RHS). LHS: two adjacent loops intersect at one base pair. RHS: two pseudoknot loops meet at two base pairs (orange)

For a sample of RNA pseudoknotted structures, the ensemble tree in our framework can still be computed. However, the structure modularity no longer holds in the pseudoknot case. The reason is that a pseudoknot loop could intersect in more than one base pair with other loops, see Fig. 17 (RHS). The fragmentation with respect to a base pair involved in a pseudoknot could affect several loops, each contributing to the free energy. The change of loop-based energy could lead to splits folding into a different configuration compared to the full transcript. Nevertheless, it would be interesting to find out other experimental methods to facilitate our framework for RNA pseudoknotted structures.

## Conclusion

In this paper we propose to enhance the method of identifying the target structure based on RNA probing data. To facilitate this we introduce the framework of ensemble trees in which a sample derived from the partition function of structures is recursively split via queries using information theory. Each query is answered based on either RNA folding data in combination with chemical probing, employing modularity of RNA structures, or, alternatively, directly using experimental methods [29, 30]. The former type of inference can be viewed as a kind of localization of probing data, relating local to global data by means of structural modularity. We show that within this framework it is possible to identify the target with high fidelity and that this identification requires a small number of base pairs to be queried. In particular we present that, for the Boltzmann ensembles incorporating probing data via pseudo-energies, the probability of the ensemble tree identifying the correct leaf that contains the target is greater than $90\%$, see “Target identification” section.

## Figures and Tables

**Fig. 18:**
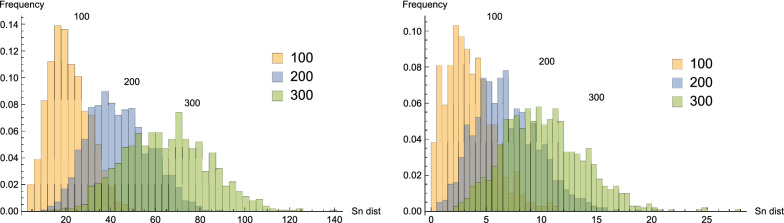
The distributions of the signature distances $d_{\text {sn}}(s,\Omega )$ and $d_{\text {sn}}(s,\Omega _{\text {probe}})$ between the target *s* and two types of Boltzmann ensembles. LHS utilizes unrestricted samples $\Omega$ of structures, and RHS uses samples $\Omega _{\text {probe}}$ incorporating probing data simulated from the signature of the target structure *s*

**Fig. 19:**
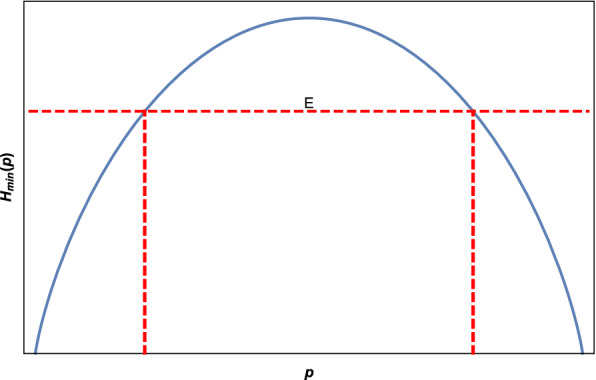
The graph of $H_{\min }(p)$ as a function of *p*

## Data Availability

Source code of the implementation of our algorithm is available from https://github.com/GaussBackyard/RNAStructureIdentifier.

## Appendices

### Appendix A: RNA secondary structures

Most computational approaches of RNA structure prediction reduce to a class of coarse grained structures, i.e. the RNA secondary structures [1, 35–38]. These are contact structures via abstracting from the actual spatial arrangement of nucleotides. An RNA secondary structure can be represented as a *diagram*, a labeled graph over the vertex set $\{1, \dots , n\}$ whose vertices are arranged in a horizontal line and arcs are drawn in the upper half-plane. Clearly, vertices correspond to nucleotides in the primary sequence and arcs correspond to the Watson-Crick **A-U**, **C-G** and wobble **U-G** base pairs. Two arcs $(i_1,j_1)$ and $(i_2,j_2)$ form a pseudoknot if they cross, i.e. the nucleotides appear in the order $i_1<i_2<j_1<j_2$ in the primary sequence. An RNA *secondary structure* is a diagram without pseudoknots.

We define two distances for comparing two structures, the base-pair and signature distances.

The base-pair distance utilizes a representation of a secondary structure *s* as a bit string ${\mathbf {b}}(s)=b_1b_2\ldots b_{l}$, where *l* denotes the number of all possible base pairs, and $b_k$ is a bit. Given the arc set *E* equipped with the lexicographic order, we define $b_k=1$ if *s* contains the *k*-th base pair in *E*, otherwise $b_k=0$. The *base-pair distance*$d_{\text {bp}}(s,s')$ between two structures *s* and $s'$ is the Hamming distance between their corresponding bit strings ${\mathbf {b}}(s)$ and ${\mathbf {b}}(s')$ .

The *0-1 signature* (or simply signature) of a structure *s*, is a vector ${\mathbf {q}}(s)=(q_1,q_2,\ldots ,q_n)$, where $q_k=0$ when the *k*-th base is unpaired in *s*, otherwise $q_k=1$. The *signature distance*$d_{\text {sn}}(s,s')$ between two structures *s* and $s'$ is defined as the Hamming distance between their corresponding 0-1 signatures ${\mathbf {q}}(s)$ and ${\mathbf {q}}(s')$. By construction, the 0-1 signature of a secondary structure mimics its probing signals, and the signature distance measures the similarity between the probing profiles of two structures. By observing that each bit corresponds to two base-pairing end, we derive $d_{\text {sn}}(s,s') \le 2 d_{\text {bp}}(s,s')$ for any *s* and $s'$.

### Appendix B: Energy model

Computational prediction of RNA secondary structures is mainly driven by loop-based energy models [25, 26]. The key assumption of these approaches is that the free energy *E*(*s*) of an RNA secondary structure *s*, is estimated by the sum of energy contributions *E*(*L*) from its individual loops *L*, $E(s)=\sum _{L} E(L)$.

According to thermodynamics, the free energy reflects not only the overall stability of the structure, but also its probability appearing in thermodynamic equilibrium. This leads to the Boltzmann sampling [4, 12] of secondary structure based on their equilibrium probabilities, whose computation can be facilitated by the partition function [3].

In this model, the energy contribution of a base pair depends on the two adjacent loops that intersect at the base pair, see Fig. 17 (LHS). Note that, in a pseudoknot, since two adjacent loops may intersect at several base pairs, and thus the energy contribution of a base pair could affect several loops, see Fig. 17 (RHS).

### Appendix C: Chemical probing

The basic idea of RNA structure probing is that chemical probes react differently with paired or unpaired nucleotides. More reactive regions of the RNA are likely to be single stranded and less reactive regions are likely to be base paired. Thus every nucleotide in a folded RNA sequence can be assigned a reactivity score, which depends on the type of chemical or enzymatic footprinting experiments and the strength of the reactivity. It is rarely of absolute certainty, whether or not a specific position is unpaired, or paired; instead, the method produces a probability. The probing data thus produce a vector of probabilities. Several competing methods have been developed to convert the footprinting data for each nucleotide into a probability. Due to its ambiguity, probing data has been further incorporated into RNA folding algorithms by adding a pseudo-energy term, $\Delta G(s)$, to the free energy [7, 10, 11], i.e.

$$\begin{aligned} E_{\text {probe}}(s)= E(s)+ \Delta G(s). \end{aligned}$$

This term engages in the folding process as follows: while positions where structure prediction and experiment data agree with each other are rewarded by a negative pseudo-energy, mismatching locations receive a penalty by way of a positive term. This is tantamount to shifting the partition function in such a way that the equilibrium distribution of structures in $\Omega _{\text {probe}}$ favors those that agree with the data.

### Appendix D: *q*-Boltzmann sampler

Here we incorporate the signature of a target via restricted Boltzmann sampling structures with the signature distance filtration.

We first analyze the signature distances in two classes of Boltzmann samples, one being unrestricted, $\Omega$, and the other being restricted $\Omega _{\text {probe}}$ that incorporates simulated probing data via pseudo-energies. The target structure is randomly selected from the unrestricted sample, and probing data is simulated from the signature of the target by a binary model. That is, the reactivity is set to 0.1 when a nucleotide is unpaired in the target, and to 0.7 when it is paired. These values are computed from the mean of collected SHAPE data among both paired and unpaired nucleotides in *E. coli* sequences [8]. According to [7], if SHAPE reactivity is 0.36, which lies in the middle of 0.1 and 0.7, there is no added pseudo-energy, i.e., $\Delta G=0$.

For both types of samples, the distribution of the signature distance between the target *s* and the ensemble is approximately normal, Fig. 18. The means and variances of the normalized signature distance are shown in Table 6. It shows that, while the average signature distance between the target and the unrestricted sampled structure is around 0.21*n*, integrating the signature of the target reduces the distance to 0.03*n*. This indicates that the incorporation of the signature improves the accuracy of the Boltzmann sampler identifying the target.

**Table 6:** The means and variances of the normalized signature distances between the target *s* and the Boltzmann samples $\Omega$, $\Omega _{\text {probe}}$ or $\Omega ^q$

	$n=100$	$n=200$	$n=300$
$d_{\text {sn}}(s,\Omega )/n$	$0.214 \pm 0.088$	$0.219\pm 0.068$	$0.217 \pm 0.063$
$d_{\text {sn}}(s,\Omega _{\text {probe}})/n$	$0.035 \pm 0.021$	$0.034\pm 0.015$	$0.034\pm 0.012$
$d_{\text {sn}}(s,\Omega ^{0.05})/n$	$0.031 \pm 0.008$	$0.038\pm 0.012$	$0.037\pm 0.018$
$d_{\text {sn}}(s,\Omega ^{0.1})/n$	$0.074 \pm 0.014$	$0.080\pm 0.015$	$0.087 \pm 0.010$
$d_{\text {sn}}(s,\Omega ^{0.15})/n$	$0.098 \pm 0.021$	$0.116 \pm 0.018$	$0.123 \pm 0.011$
$d_{\text {sn}}(s,\Omega ^{0.20})/n$	$0.127 \pm 0.034$	$0.144 \pm 0.027$	$0.157 \pm 0.020$
$d_{\text {sn}}(s,\Omega ^{0.25})/n$	$0.144 \pm 0.043$	$0.167 \pm 0.038$	$0.180 \pm 0.029$

For $\Omega$ and $\Omega _{\text {probe}}$, we utilize the same Boltzmann samples as described in Fig. 18. Values following the ± symbols are the standard deviation of the sampling errors

The above analysis motivates us to introduce a *q*-Boltzmann sampler for structures with signature distance filtration. For any fraction $q\in (0,1)$, let $\Omega ^q$ denote the restricted Boltzmann ensemble of structures having signature distance to the target at most $q\cdot n$, i.e., $\Omega ^q=\{ s'| d_{\text {sn}}(s',s) \le q\cdot n \}$. The enhanced Boltzmann sampling can be implemented by partition function [3] and stochastic backtracking technique [4], with the augmentation via an additional index recording the signature distance. A complete description of the new sampler will be provided in a future publication. The constraint on the signature distance changes the equilibrium distribution of structures via eliminating those that are inconsistent with signature over certain ratio *q*. Table 6 shows the means and variances of the normalized signature distance for $\Omega ^q$. In particular, we observe that Boltzmann samples $\Omega _{\text {probe}}$ incorporating the probing data via pseudo-energies behave similarly as *q*-samples having $q=0.05$.

### Appendix E: State-of-the-art experimental approaches

Determination of base pairs is a fundamental and longstanding problem in RNA biology. A large variety of experimental approaches have been developed to provide reliable solutions to the problem, such as X-ray crystallography, nuclear magnetic resonance (NMR), cryogenic electron microscopy (cryo-EM), chemical and enzymatic probing, cross-linking [39–42]. Each method has certain strengths and limitations. In particular, chemical probing, as one of the most widely accepted experiments, allows to detect RNA duplexes in vitro and in vivo, and has been combined with high-throughput sequencing to facilitate large-scale analysis on lncRNAs [42]. Thus, in the following, we focus on determining the queried base pairs via chemical probing.

Chemical probing data is *one-dimensional*, i.e. it does not specify base pairing partners. Thus probing data itself does not directly detect base pairings, and any structure information can only be *inferred* based on compatibility with probing data. Two strategies of structural inference have been developed, correlation analysis and mutate-and-map. [29] introduce PAIR-MaP, which utilizes mutational profiling as a sequencing approach and correlation analysis on profiles. The authors claim that PAIR-MaP provides around 0.90 accuracy of structure modeling (on average, sensitivity 0.96 and false discovery rate 0.03). [30] introduce M2-seq, a mutate-and-map approach combined with next generation sequencing, which recovers duplexes with a low false discovery rate ($<0.05$).

### Appendix F: Structural entropy

#### **Proposition 4**

*Let*$\Omega '$*be a sample of size**N**and*$s\in \Omega '$*be a structure having probability*$p_0$. *Then the structural entropy of*$\Omega '$*is bounded by*

$$\begin{aligned} H_{\min }(p_0) \le H(\Omega ') \le H_{\max } (p_0), \end{aligned}$$

*where*

$$\begin{aligned} H_{\min }(p_0)&=-p_0 \log _2 p_0 -(1-p_0) \log _2 (1-p_0),\\ H_{\max }(p_0)&=-p_0 \log _2 p_0+(1-p_0) \log _2 N. \end{aligned}$$

#### *Proof*

By construction, the multiplicity of *s* in $$\Omega '$$ is given by $$p_0^N=\lfloor p_0 N\rfloor $$. Since the function $$-x \log _2 x$$ is for $$x>0$$ concave, the structural entropy is maximal in case of all remaining $$N-p_0^N $$ structures being distinct, i.e. each occurs with probability $$(1-p_0)/(N-p_0^N)=1/N$$. Therefore

$$\begin{aligned} H_{\max }(p_0)&=-p_0 \log _2 p_0 -\sum _{N-p_0^N} \frac{1}{N}\log _2 \frac{1}{N}\\&=-p_0 \log _2 p_0+(1-p_0) \log _2 N. \end{aligned}$$

On the other hand, the minimum is achieved when all remaining structures are the same. Thus $H_{\min }(p_0) =-p_0 \log _2 p_0 -(1-p_0) \log _2 (1-p_0)$. $\square$

Now we prove Proposition 1.

#### *Proof of Proposition 1*

Let $s_0$ be the structure having the highest probability $p_0$ in $\Omega '$. By Proposition 4, we have

3 $$\begin{aligned} H_{\min }(p_0) \le E. \end{aligned}$$

Inspection of the graph of $H_{\min }(p)$ as a function of *p*, we conclude, that for $E<1$, two solutions of the equation $H_{\min }(p)=E$ exist, one being for $f(E)> 0.5$ and the other for $g(E) < 0.5$, see Fig. 19. In case of $E=1$, we have the unique solution, $f(E)=g(E)=0.5$. Since $H_{\min }(p)$ is monotone over [0, 0.5] and [0.5, 1], inequality (3) implies

$$ p_0 \ge f(E) \quad \text { or}\quad p_0 \le g(E). $$

We shall proceed by excluding $$p_0 \le g(E)$$. A contradiction, suppose that $$p_0<0.5$$ and that structures in $$\Omega '$$ are arranged in descending order according to their probabilities $$p_i $$ for $$i=0,1,\ldots , k$$. Since each structure in $$\Omega '$$ has probability smaller than 0.5, the sample $$\Omega '$$ contains at least three different structures, i.e. $$k\ge 2$$. By construction, we have $$p_i \le p_0 <0.5 $$. Now we consider the following optimization problem

$$\begin{aligned} \min _{p_i} \quad&\sum _{i=0}^k p_i\log _2 p_i \\ \text {s.t.} \quad&\sum _{i=0}^k p_i=1 \\&0\le p_k \le p_{k-1}\le \cdots \le p_0 \le 0.5. \\ \end{aligned}$$

We inspect that the multivariate function $$\sum _{i=0}^k p_i\log _2 p_i$$ reaches its minimum 1 only for $$ p_0=p_1=0.5$$ and $$p_i=0$$ for $$i \ge 2$$. In the case of $$ p_0<0.5$$, the minimum cannot be reached and we arrive at some $$E>1$$, in contradiction to our assumption $$E\le 1$$. Therefore $$p_0 \ge f(E)$$ is the only possible scenario, i.e., $$\Omega '$$ contains a distinguished structure with probability at least *f*(*E*). $$\square $$

### Appendix G: Information theory

Here we will provide proofs of the information-theoretic results on the Boltzmann ensemble of secondary structures. In particular, we point out that these results on the Boltzmann ensemble hold in the more general setup, i.e., discrete probability spaces. Let $(\Omega ,{\mathcal {P}}(\Omega ),p)$ be a discrete probability space consisting of the sample space $\Omega$, its power set ${\mathcal {P}}(\Omega )$ as the $\sigma$-algebra and the probability measure *p*. We shall refer to the space as $\Omega$. The *Shannon entropy* of $\Omega$ is given by

$$\begin{aligned} H(\Omega ) = -\sum _{s\in \Omega } p(s) \log _2 p(s), \end{aligned}$$

where the units of *H* are in bits.

A *feature**X* is a discrete random variable defined on $\Omega$. Assume that *X* has a finite number of values $x_1,x_2,\ldots ,x_k$. Set $q_i={\mathbb {P}}(X=x_i)$. The *Shannon entropy**H*(*X*) of the feature *X* is given by

$$\begin{aligned} H(X)&= -\sum _{i} q_i \log _2 q_i. \end{aligned}$$

In particular, the values of *X* define a partition of $\Omega$ into disjoint subsets $\Omega _i=\{s\in \Omega :X(s)=x_i\}$, for $1\le i \le k$. This further induces *k* spaces $(\Omega _i,{\mathcal {P}}(\Omega _i),p_i)$, where the induced distribution is given by

$$\begin{aligned} p_i(s)=\frac{p(s)}{q_i} \quad \text { for } s\in \Omega _i, \end{aligned}$$

and $q_i$ denotes the probability of *X* having value $x_i$ and is given by

$$\begin{aligned} q_i ={\mathbb {P}}(X=x_i)= \sum _{s\in \Omega _i} p(s). \end{aligned}$$

Let $H(\Omega |X)$ denote the *conditional entropy* of $\Omega$ given the value of *X*. The entropy $H(\Omega |X)$ gives the expected value of the entropies of the conditional distributions on $\Omega$, averaged over the conditioning feature *X* and can be computed by

$$\begin{aligned} H(\Omega |X)= \sum _i q_i H(\Omega _i). \end{aligned}$$

Then the *entropy reduction*$R(\Omega ,X)$ of $\Omega$ for feature *X* is the difference between the *a priori* Shannon entropy $H(\Omega )$ and the conditional entropy $H(\Omega |X)$, i.e.

$$\begin{aligned} R(\Omega ,X)= H(\Omega )-H(\Omega |X). \end{aligned}$$

The entropy reduction indicates the change on average in information entropy from a prior state to a state that takes some information as given.

Now we prove Propositions 2 and 3.

#### *Proof of Proposition 2*

$$\begin{aligned} H(\Omega |X)&= \sum _i q_i H(\Omega _i)\\&= - \sum _i q_i \sum _{s\in \Omega _i} p_i(s) \log _2 p_i(s)\\&=- \sum _i q_i \sum _{s\in \Omega _i} \frac{p(s)}{q_i} \log _2\frac{p(s)}{q_i}\\&=- \sum _i \sum _{s\in \Omega _i} p(s) ( \log _2 p(s)- \log _2 q_i)\\&= - \sum _i \sum _{s\in \Omega _i} p(s) \log _2 p(s)+ \sum _i \log _2 q_i \sum _{s\in \Omega _i} p(s) \\&=-\sum _{s\in \Omega } p(s) \log _2 p(s)+\sum _{i} q_i \log _2 q_i\\&=H(\Omega ) -H(X). \end{aligned}$$

Therefore Eq. (2) follows. $\square$

#### *Proof of Proposition 3*

By definition,

$$ {\mathbb {P}}(\Omega _1^{i,j})=\sum _{s \in \Omega _1^{i,j}} p(s) ={\mathbb {P}}(X_{i,j}(s)=1) =p_{i,j}.$$

Similarly, we have ${\mathbb {P}}(\Omega _0^{i,j})=1-p_{i,j}$. Thus $|{\mathbb {P}}(\Omega _0^{i,j}) -{\mathbb {P}}(\Omega _1^{i,j}) |=|1-2 p_{i,j}|$ is strictly decreasing on $p_{i,j} \in [0,1/2]$ and strictly increasing on [1/2, 1]. Meanwhile, the function $H(X_{i,j})=-p_{i,j} \log _2 p_{i,j} -(1-p_{i,j} ) \log _2 (1-p_{i,j} )$ is strictly increasing on $p_{i,j} \in [0,1/2]$ and symmetric with respect to $p_{i,j} =1/2$. Therefore, $|{\mathbb {P}}(\Omega _0^{i,j}) -{\mathbb {P}}(\Omega _1^{i,j}) |$ reaches its minimum when $H(X_{i,j})$ has the maximum value, that is, $X_{i_0,j_0}$.

Assertion (2) follows directly from Proposition 2. $\square$

Given two features $X_1$ and $X_2$, we can partition $\Omega$ either first by $X_1$ and subsequently by $X_2$, or first by $X_2$ and then by $X_1$, or just by a pair of features $(X_1,X_2)$. In the following, we will show that all three approaches provide the same entropy reduction of $\Omega$.

Before the proof, we define some notations. The joint probability distribution of a pair of features $$(X_1,X_2)$$ is given by $$q_{i_1,i_2}= {\mathbb {P}}(X_1=x^{(1)}_{i_1},X_2=x^{(2)}_{i_2})$$, and the marginal probability distributions are given by $$q^{(1)}_{i_1}={\mathbb {P}}(X_1=x^{(1)}_{i_1})$$ and $$q^{(2)}_{i_2}={\mathbb {P}}(X_2=x^{(2)}_{i_2})$$. Clearly, $$\sum _{i_1}q_{i_1,i_2}=q^{(2)}_{i_2} $$ and $$\sum _{i_2}q_{i_1,i_2}=q^{(1)}_{i_1} $$. The *joint entropy*$$H (X_1,X_2) $$ of a pair $$(X_1,X_2)$$ is defined as

$$\begin{aligned} H (X_1,X_2)= -\sum _{i_1} \sum _{i_2} q_{i_1,i_2} \log _2 q_{i_1,i_2}. \end{aligned}$$

The *conditional entropy*$H(X_2|X_1)$ of a feature $X_2$ given $X_1$ is defined as the expected value of the entropies of the conditional distributions $X_2$, averaged over the conditioning feature $X_1$, i.e.

$$\begin{aligned} H(X_2|X_1)= \sum _{i_1} {\mathbb {P}}(X_1=x^{(1)}_{i_1}) H(X_2|X_1=x^{(1)}_{i_1}). \end{aligned}$$

#### **Proposition 5**

(Chain rule, [43])

4 $$\begin{aligned} H(X_1,X_2)=H(X_1)+ H(X_2|X_1). \end{aligned}$$

#### **Proposition 6**

*Let*$R(\Omega ,X_1,X_2)$*denote the**entropy reduction**of*$\Omega$*first by the feature*$X_1$*and then by the feature*$X_2$, *and*$R(\Omega ,(X_1,X_2))$*denote the**entropy reduction**of*$\Omega$*by a pair of features*$(X_1,X_2)$. *Then*

5 $$\begin{aligned} R(\Omega ,X_1,X_2) = R(\Omega ,(X_1,X_2)). \end{aligned}$$

#### *Proof*

By Proposition 2, we have

$$\begin{aligned} R(\Omega ,X_1)&= H(X_1),\\ R(\Omega ,(X_1,X_2))&= H(X_1,X_2). \end{aligned}$$

Let $\Omega _{i_1}$ denote the spaces obtained by partitioning $\Omega$ via $X_1$, i.e. $\Omega _{i_1}=(\Omega _{i_1},{\mathcal {P}}(\Omega _{i_1}),p_{i_1})$, where $\Omega _{i_1}=\{s\in \Omega :X_1(s)=x^{(1)}_{i_1}\}$, and

$$\begin{aligned} p_{i_1}(s)=\frac{p(s)}{q^{(1)}_{i_1}}, \quad \text { for } s\in \Omega _{i_1}, \end{aligned}$$

where $q^{(1)}_{i_1}={\mathbb {P}}(X_1=x^{(1)}_{i_1})$. Then the space $\Omega _{i_1}$ is further partitioned into $\Omega _{i_1,i_2}$ via $X_2$. That is, $\Omega _{i_1,i_2}=(\Omega _{i_1,i_2},{\mathcal {P}}(\Omega _{i_1,i_2}),p_{i_1,i_2})$, where $\Omega _{i_1,i_2}=\{s\in \Omega _{i_1}:X_2(s)=x^{(2)}_{i_2}\}$, and

$$\begin{aligned} p_{i_1,i_2}(s)=\frac{p_{i_1}(s)}{{\mathbb {P}}(X_2=x^{(2)}_{i_2} |X_1=x^{(1)}_{i_1})} = \frac{\frac{p(s)}{q^{(1)}_{i_1}} }{ \frac{q_{i_1,i_2}}{q^{(1)}_{i_1}}}= \frac{p(s)}{q_{i_1,i_2}}, \quad \text { for } s\in \Omega _{i_1,i_2}. \end{aligned}$$

The entropy reduction $R(\Omega ,X_1,X_2)$ is given by the difference between the *a priori* Shannon entropy $H(\Omega )$ and the conditional entropy $H((\Omega |X_1)|X_2)$, which is the expected value of the entropies of $\Omega _{i_1,i_2}$, weighted by the probability ${\mathbb {P}}(s\in \Omega _{i_1,i_2}) ={\mathbb {P}}(X_2=x^{(2)}_{i_2}, X_1=x^{(1)}_{i_1}) = q_{i_1,i_2}$. In view of Proposition 2, we derive

$$\begin{aligned} R(\Omega ,X_1,X_2)&= H(\Omega )-H((\Omega |X_1)|X_2)\\&= H(\Omega ) - \sum _{i_1,i_2} {\mathbb {P}}(s\in \Omega _{i_1,i_2}) H(\Omega _{i_1,i_2})\\&= H(\Omega ) +\sum _{i_1,i_2} {\mathbb {P}}(s\in \Omega _{i_1,i_2}) \sum _{s\in \Omega _{i_1,i_2} } p_{i_1,i_2}(s) \log _2 p_{i_1,i_2}(s) \\&= H(\Omega ) +\sum _{i_1,i_2}q_{i_1,i_2} \sum _{s\in \Omega _{i_1,i_2} } \frac{p(s)}{q_{i_1,i_2}} \log _2 \frac{p(s)}{q_{i_1,i_2}} \\&=H(\Omega ) + \sum _{i_1,i_2} \sum _{s\in \Omega _{i_1,i_2} } p(s) \log _2 p(s)- \sum _{i_1,i_2} \sum _{s\in \Omega _{i_1,i_2} } p(s) \log _2 q_{i_1,i_2}\\&=H(\Omega ) +\sum _{s\in \Omega } p(s) \log _2 p(s) - \sum _{i_1,i_2} \log _2 q_{i_1,i_2} \sum _{s\in \Omega _{i_1,i_2} } p(s)\\&=H(\Omega ) -H(\Omega ) - \sum _{i_1,i_2} q_{i_1,i_2} \log _2 q_{i_1,i_2}\\&= H(X_1,X_2)\\&= R(\Omega ,(X_1,X_2)). \end{aligned}$$

Eq. (5) follows. $$\square $$

The maximum entropy of an arbitrary feature is achieved when all its outcomes occur with equal probability, and this maximum value is proportional to the logarithm of the number of possible outcomes to the base 2. Thus Proposition 2 implies that the more possible outcomes a feature has, the higher entropy reduction it could possibly lead to.

Meanwhile, a feature with an arbitrary number of outcomes can be viewed as a combination of *binary features*, the ones with two possible outcomes. Even though the entropy of the combination of two features is greater than each of them, Proposition 6 shows that partitioning the space subsequently by two features has the same entropy reduction as partitioning by their combination. Therefore, instead of considering features with outcomes as many as possible, we focus on binary features.

### Appendix H: Query repeats

Here we assess the improvement of the error rates by repeating the same query twice. Let *Y* (or *N*) denote the event of the queried base pair existing (or not) in the target structure. Let *y* (or *n*) denote the event of the experiment confirming (or rejecting) the base pair. Let *nn* denote the event of two independent experiments both rejecting the base pair. Similarly, we have *yy* and *yn*. Utilizing the same sequences and structures as described in Fig. 5, we estimate the conditional probabilities ${\mathbb {P}}(n|N)\approx 0.993$ and ${\mathbb {P}}(n|Y)\approx 0.055$. The *prior probability*${\mathbb {P}}(Y)$ can be computed via the expected number $l_1$ of confirmed queried base pairs on the path, divided by the number of queries in each sample. Figure 11 displays the distribution of $l_1$ having mean around 5. Thus we adopt ${\mathbb {P}}(Y)= {\mathbb {P}}(N)=0.5$. By Bayes’ theorem, we calculate the *posterior*

$$\begin{aligned} {\mathbb {P}}(N|nn)=\frac{{\mathbb {P}}(nn|N) {\mathbb {P}}(N)}{{\mathbb {P}}(nn)}=\frac{{\mathbb {P}}(n|N)^2 {\mathbb {P}}(N)}{{\mathbb {P}}(n|N)^2 {\mathbb {P}}(N) +{\mathbb {P}}(n|Y)^2{\mathbb {P}}(Y)}, \end{aligned}$$

where ${\mathbb {P}}(nn)={\mathbb {P}}(nn|N) {\mathbb {P}}(N) + {\mathbb {P}}(nn|Y) {\mathbb {P}}(Y)$. Since two experiments can be assumed to conditionally independent given *Y* and also given *N*, we have ${\mathbb {P}}(nn|N) ={\mathbb {P}}(n|N)^2$ and ${\mathbb {P}}(nn|Y) ={\mathbb {P}}(n|Y)^2$. Similarly, we compute ${\mathbb {P}}(Y|nn)$, ${\mathbb {P}}(Y|yy)$ and ${\mathbb {P}}(Y|yn)$ etc, see Table 7. It demonstrates that, if we get the same answer to the query twice, the error rates would become significantly smaller, for example, $e_0^{[2]} =0.003$ and $e_1^{[2]} =0.00005$. In the case of mixed answers *ny* or *yn*, its probability ${\mathbb {P}}(ny)=0.0292$, i.e., it rarely happens. We would recommend a third experiment and take the majority of three answers when getting two mixed answers.

In principle, we can extend to reducing the error rates by repeating the same query *k* times. The above Bayesian argument is then generalized to sequential updating on the error rates from $$e_0$$ to $$e_0^{[k]} $$. We can show that $$e_0^{[k]}$$ and $$e_1^{[k]} $$ approach to 0, as *k* grows to infinity. In this case, the reliability of the leaf space $${\mathbb {P}}(s\in \Omega _{11}) $$ is 1, i.e. the leaf always contain the target. The fidelity of the distinguished structure $${\mathbb {P}}(s^{*}=s ) $$ increases from 70 to $$94\%$$ for sequences of length 300. To sum up, asking the same query a constant number of times significantly improves the fidelity of the leaf and the distinguished structure.

**Table 7:** The posterior probabilities after two experiments

Outcome of two experiments	*Y*	*N*
*nn*	${\mathbb {P}}(Y|nn)=0.003$	${\mathbb {P}}(N|nn)=0.997$
*yy*	${\mathbb {P}}(Y|yy)=0.99995$	${\mathbb {P}}(N|yy)=0.00005$
*ny* or *yn*	${\mathbb {P}}(Y|ny)=0.881$	${\mathbb {P}}(N|ny)=0.119$

We use the same sequences and structures as described in Fig. 5

## Footnotes

1 They are defined by $\text {FDR}=\frac{\text {FP}}{\text {TP}+\text {FP}}$ and $\text {FOR}=\frac{\text {FN}}{\text {TN}+\text {FN}},$, where TP (true positive) is the number of correctly identified base pairs, FP (false positive) is the number of incorrectly predicted pairs that do not exist in the accepted structure, TN (true negative) is the number of pairs of bases that are correctly identified as unpaired and FN (false negative) is the number of base pairs in the accepted RNA structure that are incorrectly predicted as unpaired.

2 Here $$d_{\text {bp}}(s,\Omega )= \sum _{s'\in \Omega } p(s') d_{\text {bp}}(s,s')$$.

3 consisting of *N* different structures with the uniform distribution, each structure containing only one base pair.
